# Non-covalent interactions in solid *p*-C_6_F_4_Cl_2_ and C_6_F_5_Cl†

**DOI:** 10.1039/d4ce01192a

**Published:** 2025-02-05

**Authors:** Joseph C. Bear, Alexander Rosu-Finsen, Jeremy K. Cockcroft

**Affiliations:** a School of Life Sciences, Pharmacy and Chemistry, Kingston University, Penrhyn Road Kingston upon Thames KT1 2EE UK; b Department of Chemistry, Christopher Ingold Laboratories, University College London 20 Gordon Street London WC1H 0AJ UK j.k.cockcroft@ucl.ac.uk

## Abstract

This study investigates the crystal structure and phase behaviour of two organofluorine aromatic compounds, *para*-dichlorotetrafluorobenzene (*p*-C_6_F_4_Cl_2_) and chloropentafluorobenzene (C_6_F_5_Cl), with a focus on solid-state phase transitions and non-covalent interactions. The thermal and structural properties of these compounds were investigated using a combination of differential scanning calorimetry (DSC), variable-temperature powder X-ray diffraction (VT-PXRD), and single-crystal X-ray diffraction (SXD). While *p*-C_6_F_4_Cl_2_ showed no solid-state phase transitions, C_6_F_5_Cl exhibited three solid-state phases, including a reversible solid–solid transition at low temperature and an elusive transition just below the melt. The phase II–III transition in C_6_F_5_Cl is due to a change from twofold disorder to an antiferroelectric arrangement of the molecular dipole moment. Phase II of C_6_F_5_Cl is isomorphous to the structure of *p*-C_6_F_4_Cl_2_. A comparison of the different solid-state structures of mono- and *para*-di-halide-substituted hexafluorobenzenes is given.

## Abstract 2

A solid-state study of the weak non-covalent interactions in *p*-C_6_F_4_Cl_2_ and C_6_F_5_Cl reveals complex phase behaviour in the latter. The disordered form II of C_6_F_5_Cl is isomorphous to the crystal structure of *p*-C_6_F_4_Cl_2_.

## Graphical abstract

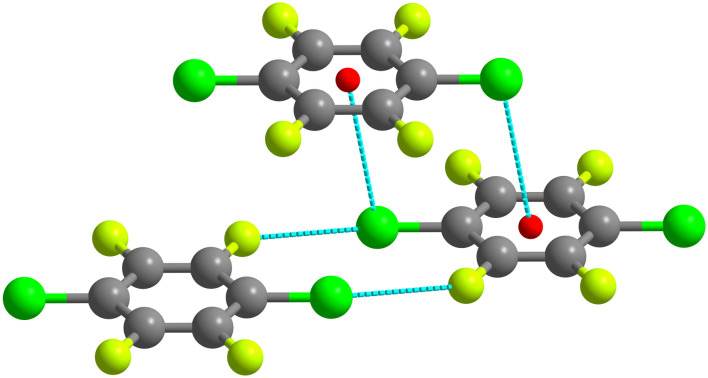

## Introduction

A co-crystal may be defined as a crystalline material made up of two or more different molecular (co-formers) that are present in a specific ratio.^1^ Co-crystals composed of charged molecular species are usually classified as salts; those comprised of one co-former and a solvent molecule such as water are usually classified as solvates. When the co-formers are ionic as in a salt, there are strong electrostatic interactions. For neutral co-formers, there can be relatively strong intermolecular forces due to non-covalent interactions such as hydrogen-donor⋯hydrogen-acceptor bonding. Weaker interactions include the recently IUPAC defined halogen bond,^2^ permanent molecular dipole and quadrupolar attractions, and van der Waals forces.^3^ The need to better understand these interactions, particularly the weaker intermolecular interactions, is of paramount importance for crystal structure prediction^4^ and the design of co-crystals with bespoke properties.^5–7^

Co-crystals themselves have been an area of intense research over the last two decades as adding a co-former to a molecule of interest can drastically alter the physical properties of the resultant crystal.^8,9^ Of particular interest in our research are co-crystals formed by electron-rich and electron-poor aromatic co-formers; the classic example being the adduct C_6_H_6_:C_6_F_6_ formed by benzene (C_6_H_6_) and hexafluorobenzene (C_6_F_6_).^10^ On mixing the two liquids in a 1 : 1 stoichiometric ratio at room temperature and pressure, the physical change is that a solid is formed. The difference in melting point between the individual components and the adduct is about 20 °C.^11^ In a related adduct, the addition of C_6_F_6_ to ferrocene Fe (C_5_H_5_)_2_ as a co-former in a 1 : 1 co-crystal alters the order-to-disorder transition temperature in the crystal by around 100 °C.^12^ Although an understanding of these differences is important for furthering fundamental science, they can be crucial in the pharmaceutical sector where the addition of something as simple as a co-former to an active pharmaceutical ingredient may affect thermal stability, hygroscopicity, organolepticity, solubility, dissolution, and bioavailability.^13,14^

The strength of non-covalent interactions in crystalline solids can be explored by varying the temperature or by perturbing the system through atomic substitution. Variable temperature studies using a combination of differential scanning calorimetry (DSC), variable-temperature powder neutron and X-ray diffraction (PND and VT-PXRD), and single-crystal X-ray diffraction (SXD) on the classic adduct C_6_H_6_:C_6_F_6_ revealed the existence of four solid-state phases, enabling their structure solution, and an analysis of the behaviour of the system with temperature.^15,16^ The structure of the highest temperature phase is determined by the quadrupolar interactions leading to close-packed stacked-columns of molecules. On lowering the temperature, the structures of the lower temperature phases are increasingly determined by the intercolumnar interactions.

The non-covalent interactions in this system can be perturbed by substitution of a hydrogen atom in C_6_H_6_ for one or more methyl (−Me) groups, *viz.* C_6_H_5_Me:C_6_F_6_, *p*-C_6_H_4_Me_2_:C_6_F_6_,^17^ and 1,3,5-C_6_H_3_Me_3_:C_6_F_6_.^18^ Studies on C_6_H_5_Me:C_6_F_6_ revealed an antiferroelectric ordering of the molecules at low temperature due to the dipole moment in C_6_H_5_Me. Increasing the temperature weakens the intermolecular interactions leading to classic twofold disorder of the C_6_H_5_Me and ultimately to sixfold disorder, similar to that observed in the adduct C_6_H_6_:C_6_F_6_. Likewise, studies on *p*-C_6_H_4_Me_2_:C_6_F_6_ show that the presence of two methyl groups locks the orientation of the molecule with respect to the column axis of the molecules in all phases. In the lowest temperature phase, the *p*-C_6_H_4_Me_2_ and C_6_F_6_ molecules exhibit an “eclipsed” arrangement but this transforms on heating to a “staggered” form. Finally, research on 1,3,5-C_6_H_3_Me_3_:C_6_F_6_ revealed three phases that are a result of a decrease in intermolecular interactions with an increase in temperature.

Alternatively, the system may be perturbed by substitution of a fluorine atom in C_6_F_6_ with a heteroatom such as Cl.^19^ In the system (C_6_H_6_:C_6_F_5_Cl), four solid-state phases were observed, three of which were structurally similar to the phases observed for C_6_H_5_Me:C_6_F_6_. In both systems, one of the co-formers has a dipole moment that can lead to antiferroelectric molecular ordering at low temperature. Lastly, substitution of atoms in both co-formers is possible as in the adduct *p*-C_6_H_4_Me_2_:C_6_F_5_H.^20^

As part of our investigations into substituted adducts, an in-depth study of the behaviour of the component molecules is required. During our study on C_6_H_6_:C_6_F_5_Cl,^19^ it became apparent that no solid structures were available for either *p*-C_6_F_4_Cl_2_ or C_6_F_5_Cl, both of which we have used in a much larger study of columnar adducts, and which is currently being prepared for publication. We note that C_6_F_5_Cl was employed as a co-former in a couple of studies by Jin *et al.*,^21^ with *p*-C_6_F_4_Cl_2_ being used recently as a co-former by Gunaga & Bryce.^22^ In the current paper we present the structure of solid *p*-C_6_F_4_Cl_2_ and the structures of two of the solid-state phases of C_6_F_5_Cl, in addition to complementary DSC and VT-PXRD data.

## Experimental

C_6_F_5_Cl (purity 99%) and *p*-C_6_F_4_Cl_2_ (purity 95%) were purchased from Sigma-Aldrich Ltd. and Manchester Organics, respectively. It was found that during re-crystallisation from C_6_F_6_, the as-supplied *p*-C_6_F_4_Cl_2_ contained a small amount of insoluble material, which was removed by filtration before use to produce a white solid (m.p. 327 K). Both compounds were analysed by differential scanning calorimetry (DSC), variable-temperature powder X-ray diffraction (VT-PXRD), and single-crystal X-ray diffraction (SXD). Detailed information on the materials, experimental methods, and instrumentation are provided in the ESI.†

## Results and discussion

**Fig. 1:**
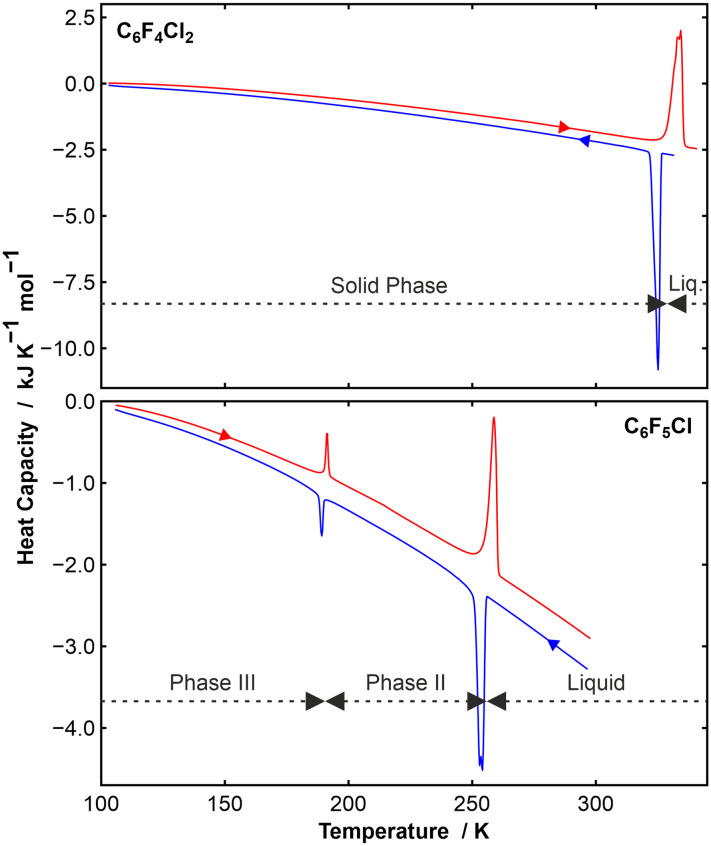
(Top) DSC data (shown endo up) on a sample of *p*-C_6_F_4_Cl_2_ showing a single solid-state phase above 100 K. The blue curve was measured on cooling and the red curve on heating. The sample froze at 325 K (Δ*H*_freeze_ = −19.2 kJ mol^−1^) and melted at 334 K (Δ*H*_fusion_ = +19.5 kJ mol^−1^); (bottom) DSC data (shown endo up) on a sample of C_6_F_5_Cl showing two solid-state phases, which we have labelled phases III and II. The sample froze at 254 K (Δ*H*_freeze_ = −6.8 kJ mol^−1^) and melted at 259 K (Δ*H*_fusion_ = +7.0 kJ mol^−1^). The III–II phase transition showed gave reproducible enthalpy values of −0.73 kJ mol^−1^ at 189 K on cooling and 0.75 kJ mol^−1^ at 191 K on heating.

The two species under scrutiny in this paper, *para*-dichlorotetrafluorobenzene (*p*-C_6_F_4_Cl_2_) and chloropenta-fluorobenzene (C_6_F_5_Cl) are aromatic organofluorine compounds, with the reported melting point of C_6_F_5_Cl being −15 °C (258 K).^23^ C_6_F_5_Cl is therefore a liquid at room temperature. By contrast, *p*-C_6_F_4_Cl_2_ is a solid with a melting point of 52 °C (325 K).^24^ Our DSC data (Fig. 1) on *p*-C_6_F_4_Cl_2_ showed a solid to liquid transition at about 330 K, but no solid-state phase transitions. By contrast, C_6_F_5_Cl showed a single solid–solid phase transition at about 190 K in addition to the solid–liquid transition at about 257 K, which are in good agreement with the literature.^23^

**Fig. 2:**
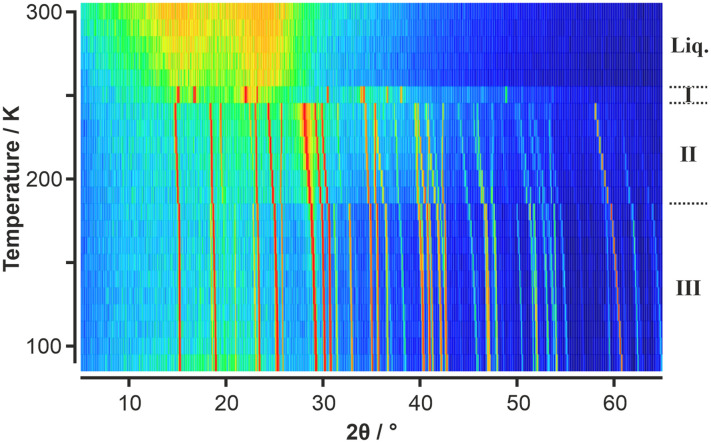
VT-PXRD data of C_6_F_5_Cl obtained on heating shown as a surface colour plot where the colour scale shows low intensities in the PXRD patterns in blue, intermediate intensities are shown in green/yellow, and high intensities in orange/red. Three solid-state phases are evident, labelled (conventionally from high to low temperature) as phases I, II, and III. The same raw data is shown as a 3-D plot in Fig. S1.†

Given the absence of any solid-state phase transitions in *p*-C_6_F_4_Cl_2_, VT-PXRD measurements on this component were deemed not necessary. However, given the observed solid–solid phase transition for C_6_F_5_Cl seen in the DSC, a VT-PXRD study was undertaken with the initial results showing three solid-state phases labelled as phases I, II, and III (Fig. 2).

Surprisingly, phase I, which was observed just below the melt on the first heating ramp, did not appear in subsequent measurements on the same sample (Fig. S2 and S3†). It is noteworthy that previous thermal measurements^23^ on C_6_F_5_Cl also observed strange solid-state phase behaviour with a phase existing over a narrow range (245 to 258 K) below the melt. Another thermal study only reported a single solid-state phase transition for C_6_F_5_Cl.^25^ Given the elusive behaviour of phase I, we undertook further VT-PXRD studies on C_6_F_5_Cl which confirmed the existence of phase I under certain conditions. We observed that capillaries which were flash frozen below 180 K resulted in “whiter-looking” samples of solid C_6_F_5_Cl (Fig. S4†) (compared to samples produced by slow cooling from the liquid) and the appearance of phase I on a heating ramp to 250 K.

**Fig. 3:**
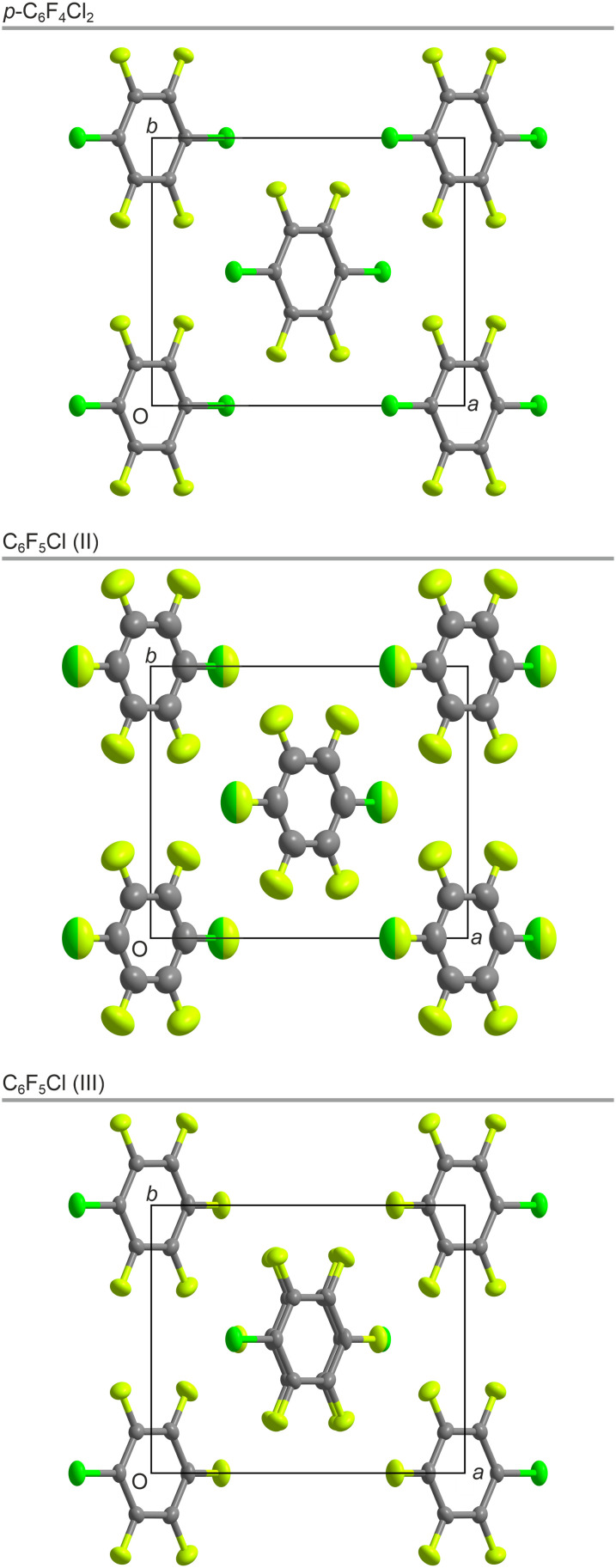
The crystal structures (C = grey, F = light green, and Cl = green) of *p*-C_6_F_4_Cl_2_ (top) at 150 K, phase II of C_6_F_5_Cl (middle) at 200 K, and phase III of C_6_F_5_Cl (bottom) at 150 K, all seen down c. F and Cl disorder in phase II is shown with two-tone ellipsoids. Centres of inversion are located at the centres of the molecules for *p*-C_6_F_4_Cl_2_ and phase II of C_6_F_5_Cl but only between molecules in phase III of C_6_F_5_Cl as a result of the doubling of the unit cell along **c**. Labelling of the atoms is shown in Fig. S5.†

**Fig. 4:**
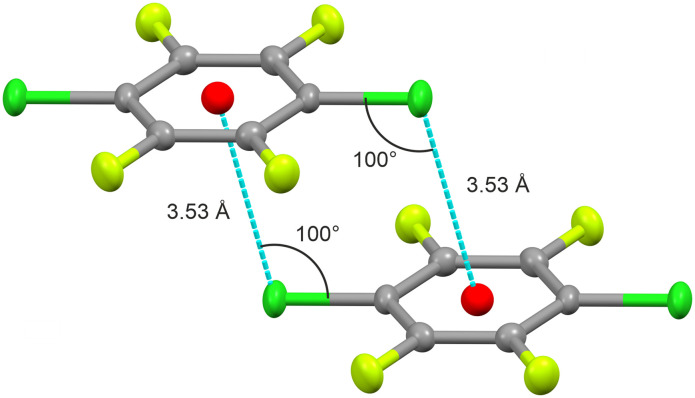
Interactions between two molecules of *p*-C_6_F_4_Cl_2_ in the solid state. Red spheres indicate the centre of mass of each molecule. Symmetry relationship between the molecules is shown in Fig. S7.†

A crystal of *p*-C_6_F_4_Cl_2_ was used for the SXD structure solution, which showed that the crystal structure is monoclinic, space group *C*2/*m*, *a* = 9.009 Å, *b* = 7.650 Å, *c* = 5.091 Å, *β* = 97.67°, with *Z*′ = ¼ (Fig. 3, top). In contrast to the solid-state structures of many simple aromatic molecules such as solid C_6_H_6_, *p*-C_6_F_4_Cl_2_ does not show a classic herringbone-type arrangement of the molecules; the normal to the planes of the aromatic rings are all aligned in the same direction (Fig. S6†). As seen in Fig. 4, the chlorine atom in one molecule approaches the π-cloud of the aromatic ring in a neighbouring molecule at a distance of 3.53 Å, the angle formed by the C–Cl bond and the Cl⋯π-cloud being less than 100°. Whether this is simply an electrostatic interaction or a halogen bond between the two is open to debate. In addition, the molecules pack in sheets such that there is close contact between the Cl atoms in one molecule and F atoms in two neighbouring molecules (Fig. S8†).

Prior to the diffraction experiments, we posed the question as to whether C_6_F_5_Cl in solid form would adopt a structure analogous to that of *p*-C_6_F_4_Cl_2_ with twofold disorder of the Cl atom or an antiferroelectric structure with dipole moment ordering of the C–Cl bond. Crystals of C_6_F_5_Cl were grown from the melt by cooling a narrow capillary *in situ* on the single-crystal X-ray diffractometer and the structure obtained using the multigrain approach adopted previously.^20^ Initially, the crystal structure was solved at 200 K in the monoclinic space-group *C*2/*m*, *a* = 9.112 Å, *b* = 7.771 Å, *c* = 4.803 Å, *β* = 95.98°, with *Z*′ = ¼ (Fig. 3, middle). Comparison with the PXRD data shows that this structure with twofold disorder of the Cl atom is the same phase II observed by VT-PXRD.

The SXD sample was then slowly cooled to 150 K and a further crystal structure was determined (Fig. 3, bottom), which corresponded to the lowest temperature phase seen in PXRD, phase III. This phase also has the monoclinic space-group *C*2/*m* (with *a* = 9.018 Å, *b* = 7.658 Å, *c* = 9.498 Å, *β* = 96.56°, and *Z*′ = ½), but with a doubled unit cell along *c* compared to phase II. Attempts to produce a single crystal in phase I by annealing just below the melt were repeatedly unsuccessful. As discussed earlier, we were only able to isolate phase I in VT-PXRD by flash cooling and reheating to just below the melt. However, this approach is incompatible with the production of crystals suitable for SXD structure determination.

As seen in Fig. 3, the structure of C_6_F_5_Cl (II) with twofold disorder of the molecules is indeed isomorphous to the solid-state structure of *p*-C_6_F_4_Cl_2_. Further, on cooling below the II–III transition temperature of about 190 K, the crystal structure changes to one in which the molecules adopt an antiferroelectric arrangement with the molecular dipole moments alternating along the *a*–*c* direction (see Fig. S9†) leading to a doubling of the unit cell along *c* and with the molecules still arranged in sheets as in *p*-C_6_F_4_Cl_2_ and C_6_F_5_Cl (II).

This formation of sheets of molecules in C_6_F_5_Cl and *p*-C_6_F_4_Cl_2_ is not seen in the solid-state structures of *p*-C_6_F_4_Br_2_ or p-C_6_F_4_I_2_, nor in C_6_F_6_ at ambient pressure.^26^ However, a recent high-pressure study by Rusek *et al.*^27^ on the latter revealed the existence of phase II in which the molecules are arranged in planes (Fig. S10†), similar to that observed in this study for *p*-C_6_F_4_Cl_2_. The nature of the F⋯F electrostatic interactions in C_6_F_6_ (II) are discussed in more detail in their paper.^27^

**Fig. 5:**
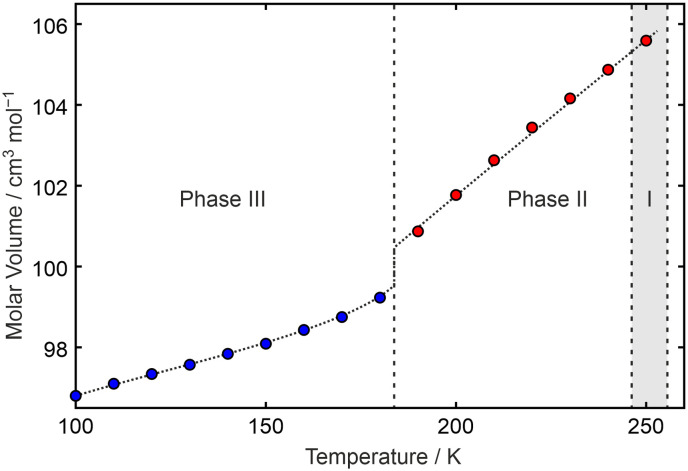
Molar volume of C_6_F_5_Cl as a function of temperature showing the step change in volume between phases II (points in red) and III (points in blue). The dotted line linking the points is a guide to the eye. Numerical values for the data points obtained from the results of LeBail fits to the repeated raw PXRD data are available in the ESI.† The grey area indicates the region where earlier DSC had reported an additional phase as seen by us under certain conditions in PXRD.

From the unit-cell information obtained from the SXD experiments, the VT-PXRD data on phases II and III could be indexed and the lattice parameters refined using the LeBail method^28^ (Table S4†). Due to sample granularity, structural information cannot be obtained from our VT-PXRD using the Rietveld method. From the refined lattice parameters, a plot of molar volume *versus* temperature is obtained (Fig. 5).

The volume plot for C_6_F_5_Cl confirms that the phase transition from an antiferroelectric structure (phase III) to one with twofold disorder of the molecules (phase II) is first order. As expected, the packing of the molecules is more efficient in phase III than in phase II. In addition, changes in the individual lattice parameters provide thermal expansion details with respect to directions that are approximately parallel and perpendicular to the plane containing the molecules (*viz.***b**, **a** − **c** (*i.e.* vector difference), and **a** + **c** (*i.e.* vector sum) for phase III and **a** − 2**c**, and **a** + 2**c** for phase II, Fig. S11†). This shows that the volume changes on heating from low temperature, *i.e.* while in phase III, are due to weakening of the intermolecular forces between layers rather than within a layer of molecules. On heating above the III–II phase-transition point, expansion occurs perpendicular to the molecular dipole-moment direction despite the two-fold disorder observed in phase II, demonstrating the strength of this electrostatic interaction over other forces (*e.g.* dispersion) holding the molecules together in the solid state.

We are unable to give a molar volume for phase I of C_6_F_5_Cl in Fig. 5 given the absence of a structure for this phase. The PXRD pattern of phase I may be partially indexed, with nearly all of the peaks accounted for in terms of *h*0*l* reflections (see Fig. S12†), but with no general *hkl* reflections to provide information on the third dimension. Crystal structure prediction may be able to suggest a structure for phase I that we have been unable to determine experimentally. The PXRD data, supplied in CIF format, may prove useful with regard to obtaining a unit-cell match.

Even prior to the discovery of X-ray diffraction, it was known that *p*-dihalobenzenes exhibit isomorphism in their solid-state structures, with both *p*-C_6_H_4_Cl_2_ and *p*-C_6_H_4_Br_2_ being reported as monoclinic in 1899.^29^ A similar isomorphism between *p*-C_6_F_4_Br_2_ and *p*-C_6_F_4_I_2_ was discussed by Pawley *et al.*,^30^ with the crystal structures being determined subsequently from SXD data.^31,32^Table 1 shows a comparison of the known crystal structures of both C_6_F_5_X and *p*-C_6_F_4_X_2_ for X = F, Cl, Br, and I.

**Table 1:** Comparison of the known crystal structures formed by perfluorobenzene and selected mono- and *para*-di-halide substituted forms (C_6_F_5_X and *p*-C_6_F_4_X_2_) determined at ambient pressure except for C_6_F_6_ (II), which is a high pressure phase. In some instances, crystal structures have been reported for more than one temperature. Crystal structures are unknown for C_6_F_5_Cl (I) and C_6_F_5_Br (III)

Compound	*T*/K	S.G.	*Z*	*Z*′	Ref.
C_6_F_6_ (I)	120	*P*2_1_/*n*	6	1½	26
C_6_F_6_ (II)	RT	*C*2/*c*	4	½	27
*p*-C_6_F_4_Cl_2_	150	*C*2/*m*	2	¼	*Hoc opus*
*p*-C_6_F_4_Br_2_	100	*P*2_1_/*c*	2	½	31
*p*-C_6_F_4_I_2_	180	*P*2_1_/*c*	2	½	32, 33
C_6_F_5_Cl (II)	200	*C*2/*m*	2	¼	*Hoc opus*
C_6_F_5_Cl (III)	150	*C*2/*m*	4	½	*Hoc opus*
C_6_F_5_Br (I)	230	*P*2_1_/*n*	8	2	19
C_6_F_5_Br (II)	220	*Pna*2_1_	12	3	19
C_6_F_5_Br (IV)	150	*P*2_1_/*c*	4	1	19
C_6_F_5_I (I)	150	*P*2_1_/*c*	4	1	34
C_6_F_5_I (II)	100	*P*2_1_/*c*	8	2	35

In contrast to the isomorphism exhibited between *p*-C_6_H_4_Cl_2_ and *p*-C_6_H_4_Br_2_, and between *p*-C_6_F_4_Br_2_ and *p*-C_6_F_4_I_2_, *p*-C_6_F_4_Cl_2_ does not show isomorphous behaviour to the latter. However, some of the non-covalent interactions observed in *p*-C_6_F_4_Cl_2_ mirror those seen in *p*-C_6_F_4_Br_2_ and *p*-C_6_F_4_I_2_, where the same interaction motif is found in the solid form of each (*cf.*Fig. 4 and S13†). By contrast, *p*-C_6_F_4_Cl_2_ exhibits an interaction not seen in *p*-C_6_F_4_Br_2_ and *p*-C_6_F_4_I_2_, which results in the molecules packing in parallel sheets (Fig. S8†), similar to that observed in the high pressure form of C_6_F_6_ (*cf.* Fig. S9†).

With regard to the mono-halide substituted derivatives (C_6_F_5_X), C_6_F_5_Br (IV) is isomorphous to form I of C_6_F_5_I, both of which show antiferroelectric ordering of the molecules. By contrast, C_6_F_5_Cl (III) shows antiferroelectric ordering of the molecules but with the molecules packing in sheets, similar to that observed in *p*-C_6_F_4_Cl_2_, and in the disordered form II of C_6_F_5_Cl.

## Conclusions

In this paper, we have successfully determined the crystal structure of solid *p*-C_6_F_4_Cl_2_ and the crystal structures of two of the solid-state phases of C_6_F_5_Cl. The crystal structure of a third elusive phase of C_6_F_5_Cl, existing just below the melt, could not be determined. In addition, we provide complementary DSC and VT-PXRD data on both compounds. In these systems, there is evidence for both Cl⋯F and Cl⋯π-cloud interactions between molecules. Additionally, the anisotropic expansion of the lattice in C_6_F_5_Cl demonstrates the influence of the molecular dipole moment on the overall structure. An understanding of these non-covalent interactions in *p*-C_6_F_4_Cl_2_ and C_6_F_5_Cl in their solid forms is important for the rationalisation of the structures of columnar adducts formed between these molecules and substituted benzenes, which will be the subject of a future paper.

## Data availability

Data supporting this article have been included as part of the ESI.† Crystal structures are available as CIF files. VT-PXRD data is available in CIF format.

## Author contributions

All authors contributed equally to the publication.

## Conflicts of interest

There are no conflicts to declare.

## Supplementary Material

CE-027-D4CE01192A-s001

CE-027-D4CE01192A-s002
