# Supplementary material for: Non-covalent interactions in solid p-C6F4Cl2 and C6F5Cl
Source: CrystEngComm. 2025 Feb 5;27(10):1386–91. doi: 10.1039/d4ce01192a (PMC11816605; doi:10.1039/d4ce01192a)
Supplement: CE-027-D4CE01192A-s001 [file CE-027-D4CE01192A-s001.pdf]

Supplementary Information (SI) for CrystEngComm.
This journal is © The Royal Society of Chemistry 2025

Supporting Information

to

Non-covalent interactions in solid *p*-$C_6F_4Cl_2$ and C_6F_5Cl

Joseph C. Bear, Alexander Rosu-Finsen, and Jeremy K. Cockcroft

Table of Contents Page No.

Additional Experimental Details

1. Sample Preparation S2

2. DSC Measurements and Analysis S2

3. VT-PXRD Measurements and Analysis S2

4. SXD Measurements and Analysis S3

List of Tables

SXD data on *p*-$C_6F_4Cl_2$ at 150 K

Table S1a. Crystal data and structure refinement S4

Table S1b. Fractional atomic coordinates and *U*(eq) for all atoms S5

Table S1c. Anisotropic displacement parameters S5

Table S1d. Selected bond lengths S5

SXD data on C_6F_5Cl (II) at 200 K

Table S2a. Crystal data and structure refinement S6

Table S2b. Fractional atomic coordinates and *U*(eq) for all atoms S7

Table S2c. Anisotropic displacement parameters S7

Table S2d. Selected bond lengths S7

SXD data on C_6F_5Cl (III) at 150 K

Table S3a. Crystal data and structure refinement S8

Table S3b. Fractional atomic coordinates and *U*(eq) for all atoms S9

Table S3c. Anisotropic displacement parameters S9

Table S3d. Selected bond lengths S9

Table S4. Summary of cell parameters and molecular volumes for C_6F_5Cl S10

List of Figures

Figure S1. First set of raw VT-PXRD data on C_6F_5Cl S11

Figure S2. Second set of raw VT-PXRD data on C_6F_5Cl S12

Figure S3. Second set of VT-PXRD data on C_6F_5Cl viewed as a surface plot S12

Figure S4. Photographs of X-ray capillaries containing C_6F_5Cl S13

Figure S5. Labels used to identify the atoms in Fig. 3. S14

Figure S6. Crystal structure of *p*-$C_6F_4Cl_2$ S15

Figure S7. Between plane interactions in *p*-$C_6F_4Cl_2$ S15

Figure S8. Within plane interactions in *p*-$C_6F_4Cl_2$ S16

Figure S9. Within plane interactions in C_6F_5Cl (III) S17

Figure S10. Within plane interactions in C_6F_6 (II) S18

Figure S11. Unit cell changes in C_6F_5Cl versus temperature S19

Figure S12. PXRD plots of phases I and II of C_6F_5Cl S20

Figure S13. Molecular interactions in *p*-$C_6F_4Br_2$ and *p*-$C_6F_4I_2$ S21

Figure S14. Crystals used in the SXD measurements on *p*-$C_6F_4Cl_2$ and C_6F_5Cl S22

Additional Experimental Details

1. Sample Preparation

C_6F_5Cl (193666-25g, MW = 202.51, purity 99%) and *p*-$C_6F_4Cl_2$ (R62299-50g, MW = 218.96, purity 95%) were purchased from Sigma-Aldrich Ltd. and Manchester Organics, respectively. Given the relatively low purity of *p*-$C_6F_4Cl_2$, about 3 g of the solid material was dissolved in excess C_6F_6 (Sigma-Aldrich Ltd., H8706-100g, MW = 186.05, purity 99%), and filtered under gravity through cotton wool to remove insoluble material. The filtrate was then left in an open bottle for the C_6F_6 to evaporate leaving behind large clear crystals of *p*-$C_6F_4Cl_2$.

2. DSC Measurements and Analyses

21.32 mg of *p*-$C_6F_4Cl_2$ was weighed (using a Mettler 5 digit balance) into an aluminium sample pan (nominal mass 50 µg), which was quickly sealed with a crimped lid. The sample pan was then loaded into a PerkinElmer DSC8000 calorimeter at +25 °C. A helium purge gas was used for all experiments (40 mL min^{-1}). For C_6F_5Cl, 16.24 mg was weighed out and the same procedure adopted.

For *p*-$C_6F_4Cl_2$, which is solid at room temperature, the sample was heated to 60 °C into the liquid phase prior to the cooling ramp at 10 °C min^{-1} to −180 °C; the sample was held isothermally for 4 min at −180 °C prior to the heating ramp to 80 °C, the latter chosen to ensure a complete transition to the liquid state. For C_6F_5Cl, the sample was initially cooled to −180 °C and then heated back to 25 °C. Prior to the cooling or heating ramp at 10 °C min^{-1}, the sample was held isothermally for 4 min.

Raw DSC data in units of mW is readily converted to DSC data in kJ K^{-1} mol^{-1} by dividing by the DSC heating rate and the molar quantity of material present. Data is shown in Fig. 1. Subsequent data analysis to determine both peak maxima and peak area used the Pyris Thermal Analysis software (version 11.1.1.0492) from PerkinElmer.

3. VT-PXRD Measurements and Analyses

A small amount of C_6F_5Cl was pipetted into the sealed end of an X-ray capillary (nominal Ø 1 mm), shaken to one end, and then carefully flame-sealed. VT-PXRD measurements were performed using a Stoe Stadi-P diffractometer equipped with a Cu anode, Ge<111> monochromator, a Dectris Mythen 1K detector, and an Oxford Instruments CryojetHT (90-500 K).

C_6F_5Cl was quenched initially at about 150 K to a white powder and then cooled *in situ* to 90 K. PXRD patterns were obtained on heating in 10 K intervals from 90 K to 300 K. The detector was scanned in 2θ from 0° to 65° in steps of 0.5° at 10 s per step, a complete scan lasting approx. 30 min; each 10 K temperature change took approx. 7-10 min and the sample was equilibrated at the set temperature for 5 min before starting the next scan. The data are shown in Figs. 2 and S1. As C_6F_5Cl showed an unexpected phase transition at about 250 K, a repeat experiment was performed 2 weeks later on the same capillary for the temperature range 100 K to 260 K (Figs. S2 and S3).

Given the difference between the two sets of data, the VT-PXRD experiments were repeated with a fresh sample. Initially, phase I of C_6F_5Cl remained elusive until the sample was flash-

cooled from the melt by pre-cooling the CryojetHT to below 150 K, and then moving it into position over the capillary, the whole procedure taking less than 10 s. This procedure resulted in a whiter looking sample (see Fig. S4) and, on heating back to 250 K, the appearance of peaks due to phase I. This procedure resulted in phase I being observed routinely. By contrast, slow cooling resulted in a greyer looking sample which became transparent close to the melt (see Fig. S4) and which did not transform into phase I on heating to the melt. Sample granularity was evident for all of the VT-PXRD measurements made, even when the sample was flash cooled.

SXD Measurements and Analysis

For *p*-$C_6F_4Cl_2$, a crystal was selected from the recrystallized material and mounted on a 0.3 mm loop from Hampton Research (see Fig. S14). The crystal was cooled to 150 K. Given that C_6F_5Cl is a liquid at room temperature, a drop of the liquid was transferred to a 0.5 mm capillary and flame sealed. Large crystals of C_6F_5Cl were grown *in situ* by repeated gentle melting and freezing of the sample close to the melting point until a dominant single crystal formed. Measurements were made on phase II of C_6F_5Cl at 200 K and on phase III at 150 K. Phase I was never observed in our SXD measurements, which is consistent with the behaviour of a sample undergoing slow cooling from the melt in VT-PXRD experiments.

SXD measurements were made using an Agilent Oxford Diffraction SuperNova equipped with a microfocus Cu Kα X-ray source and an Atlas CCD detector. Samples were cooled with a Cryojet5® developed by Oxford Instruments; the device used for these measurements is the original prototype and the Pt-resistance sensor is located in the copper-block heat exchanger and not in the nozzle of the instrument close to the sample. Thus the temperatures quoted in these SXD experiments should be treated as nominal (despite stability to much better than 0.1 °C). Total collection time (typically 2 hours to 12 hours) for full spheres of data to a resolution of 0.84 Å varied depending on size and quality of crystal, and sample temperature. Data were collected using 1° scan frames in ω and reduced using the CrysAlis^{Pro} software package (version 1.171.40.57a from Rigaku Oxford Diffraction[1]). The structures were solved using intrinsic phasing by ShelXT[2] and refined by least-squares using ShelXL 2014[3] within the Olex2 program suite.[4] For phase II of C_6F_5Cl, 50:50 disorder of F(1) and Cl(1) is imposed by inversion symmetry; in addition, the atomic coordinates and anisotropic displacement parameters for F(1) and Cl(1) were constrained to be equal. Crystal structures are illustrated with the program Mercury from CCDC with thermal ellipsoids shown at 50% probability.[5]

[1] https://www.rigaku.com/en/products/smc/crysalis

[2] G. M. Sheldrick, *Acta Crystallogr., Sect. A: Found. Adv.*, 2015, **71**, 3–8.

[3] G. M. Sheldrick, *Acta Crystallogr., Sect. C: Struct. Chem.*, 2015, **71**, 3–8.

[4] O. V. Dolomanov, L. J. Bourhis, R. J. Gildea, J. A. K. Howard and H. Puschmann, *J. Appl. Crystallogr.*, 2009, **42**, 339-341.

[5] C. F. Macrae, I. Sovago, S. J. Cottrell, P. T. A. Galek, P. McCabe, E. Pidcock, M. Platings, G. P. Shields, J. S. Stevens, M. Towler and P. A. Wood, *J. Appl. Crystallogr.*, 2020, **53**, 226–235.

Table S1a. Crystal data and structure refinement for *p*-$C_6F_4Cl_2$ at 150 K.

Identification code	exp_2986
Empirical formula	$C_6Cl_2F_4$
Formula weight	218.96
Temperature / K	150
Crystal system	monoclinic
Space group	$C2/m$
a / Å	9.0094(4)
b / Å	7.6502(4)
c / Å	5.0908(2)
α / °	90
β / °	97.673(4)
γ / °	90
Volume / Å^3	347.74(3)
Z	2
ρ_{calc} / g cm^{-3}	2.091
μ / mm^{-1}	8.634
F(000)	212.0
Crystal size / mm^3	0.283 × 0.251 × 0.194
Radiation	Cu Kα (λ = 1.54184 Å)
2θ range for data collection / °	15.252 to 146.282
Index ranges	$-11 \le h \le 11$, $-8 \le k \le 9$, $-6 \le l \le 6$
Reflections collected	1936
Independent reflections	368 [R_{int} = 0.0208, R_{sigma} = 0.0103]
Data/restraints/parameters	368/0/31
Goodness-of-fit on F^2	1.168
Final R indexes [$I \ge 2\sigma(I)$]	R_1 = 0.0220, wR_2 = 0.0596
Final R indexes [all data]	R_1 = 0.0221, wR_2 = 0.0596
Largest diff. peak/hole / e Å^{-3}	0.26/−0.17

Table S1b. Fractional atomic coordinates and equivalent isotropic displacement parameters for *p*-$C_6F_4Cl_2$ at 150 K. U_{eq} is defined as ⅓ of the trace of the orthogonalised U_{ij} tensor.

Atom	*x*	*y*	*z*	*U*(eq) / Å^2
Cl(1)	0.23788(5)	0	−0.38996(9)	0.0297(2)
F(1)	0.10153(10)	−0.30772(11)	−0.16995(18)	0.0314(3)
C(1)	0.1071(2)	0	−0.1751(4)	0.0212(4)
C(2)	0.05267(15)	−0.15523(18)	−0.0866(3)	0.0223(3)

Table S1c. Anisotropic displacement parameters for *p*-$C_6F_4Cl_2$ at 150 K. The anisotropic displacement factor exponent has the form: $-2\pi^2[h^2a^{*2}U_{11}+2hka^*b^*U_{12}+\ldots]$.

Atom	U_{11} / Å^2	U_{22} / Å^2	U_{33} / Å^2	U_{23} / Å^2	U_{13} / Å^2	U_{12} / Å^2
Cl(1)	0.0260(3)	0.0409(4)	0.0246(3)	0	0.0125(19)	0
F(1)	0.0364(5)	0.0227(5)	0.0372(5)	−0.0058(4)	0.0122(4)	0.0050(4)
C(1)	0.0186(8)	0.0275(10)	0.0182(9)	0	0.0050(7)	0
C(2)	0.0227(6)	0.0210(7)	0.0229(7)	−0.0028(5)	0.0027(5)	0.0025(5)

Table S1d. Selected bond lengths for *p*-$C_6F_4Cl_2$ at 150 K.

Atom — Atom	Length / Å	Atom — Atom	Length / Å
Cl(1) — C(1)	1.7110(19)	C(1) — C(2)	1.3831(17)
F(1) — C(2)	1.3359(16)	C(2) — C(2)^2	1.379(3)
C(1) — C(2)^1	1.3831(17)		

^{1} $x, -y, z$; ^{2} $-x, y, -z$

Table S2a. Crystal data and structure refinement for C_6F_5Cl (II) at 200 K.

Identification code	exp_2989
Empirical formula	C_6ClF_5
Formula weight	202.51
Temperature / K	200
Crystal system	monoclinic
Space group	$C2/m$
a / Å	9.1116(6)
b / Å	7.7713(6)
c / Å	4.8027(4)
α / °	90
β / °	95.977(7)
γ / °	90
Volume / Å^3	338.23(4)
Z	2
ρ_{calc} / g cm^{-3}	1.988
μ / mm^{-1}	5.481
$F(000)$	196.0
Crystal size / mm^3	0.961 × 0.365 × 0.357
Radiation	Cu Kα (λ = 1.54184 Å)
2θ range for data collection / °	19.592 to 145.784
Index ranges	$-11 \leq h \leq 11$, $-9 \leq k \leq 9$, $-5 \leq l \leq 5$
Reflections collected	2160
Independent reflections	359 [R_{int} = 0.1277, R_{sigma} = 0.0518]
Data/restraints/parameters	359/0/32
Goodness-of-fit on F^2	1.084
Final R indexes [$I \geq 2\sigma(I)$]	R_1 = 0.0694, wR_2 = 0.1861
Final R indexes [all data]	R_1 = 0.0725, wR_2 = 0.1889
Largest diff. peak/hole / e Å^{-3}	0.20/−0.33

Table S2b. Fractional atomic coordinates and equivalent isotropic displacement parameters for C_6F_5Cl (I) at 200 K. U_{eq} is defined as ⅓ of the trace of the orthogonalised U_{ij} tensor.

Atom	*x*	*y*	*z*	**U(eq) / Å^2**
Cl(1)/F(1)†	0.2292(2)	0	−0.3783(4)	0.1069(14)
F(2)	0.1038(3)	−0.3031(4)	−0.1829(7)	0.1096(16)
C(1)	0.1068(5)	0	−0.1789(11)	0.0731(16)
C(2)	0.0537(4)	−0.1523(5)	−0.0897(8)	0.0750(14)

† Occupancy constraints: Cl(1) = F(1) = 0.5

Table S2c. Anisotropic displacement parameters for C_6F_5Cl (I) at 200 K. The anisotropic displacement factor exponent has the form: $-2\pi^2[h^2a^{*2}U_{11}+2hka^*b^*U_{12}+\ldots]$.

Atom	U_{11} / Å^2	U_{22} / Å^2	U_{33} / Å^2	U_{23} / Å^2	U_{13} / Å^2	U_{12} / Å^2
Cl(1)/F(1)	0.0875(18)	0.1560(3)	0.0810(18)	0	0.0265(11)	0
F(2)	0.097(2)	0.0788(19)	0.149(3)	−0.0290(15)	−0.0064(17)	0.0241(13)
C(1)	0.055(3)	0.091(4)	0.073(3)	0	0.003(2)	0
C(2)	0.066(2)	0.067(2)	0.089(3)	−0.0095(16)	−0.0061(17)	0.0077(14)

Table S2d. Selected bond lengths for C_6F_5Cl (I) at 200 K.

Atom — Atom	Length / Å	Atom — Atom	Length / Å
Cl(1)/F(1) — C(1)	1.544(6)	C(1) — C(2)	1.364(5)
F(2) — C(2)	1.351(4)	C(2) — C(2)^2	1.370(9)
C(1) — C(2)^1	1.364(5)		

^{1} x, 1−y, z; ^{2} 1−x, y, 1−z

Table S3a. Crystal data and structure refinement for C_6F_5Cl (III) at 150 K.

Identification code	exp_2991
Empirical formula	C_6ClF_5
Formula weight	202.51
Temperature / K	150
Crystal system	monoclinic
Space group	$C2/m$
a / Å	9.0176(3)
b / Å	7.6579(3)
c / Å	9.4977(4)
α / °	90
β / °	96.562(3)
γ / °	90
Volume / Å^3	651.58(4)
Z	4
ρ_{calc} / g cm^{-3}	2.064
μ / mm^{-1}	5.690
F(000)	392.0
Crystal size / mm^3	0.947 × 0.358 × 0.334
Radiation	Cu Kα (λ = 1.54184 Å)
2θ range for data collection / °	9.374 to 146.008
Index ranges	$-11 \leq h \leq 11$, $-9 \leq k \leq 9$, $-11 \leq l \leq 11$
Reflections collected	3896
Independent reflections	699 [R_{int} = 0.0472, R_{sigma} = 0.0210]
Data/restraints/parameters	699/0/61
Goodness-of-fit on F^2	1.083
Final R indexes [$I \geq 2\sigma(I)$]	R_1 = 0.0343, wR_2 = 0.0934
Final R indexes [all data]	R_1 = 0.0357, wR_2 = 0.0944
Largest diff. peak/hole / e Å^{-3}	0.23/−0.28

Table S3b. Fractional atomic coordinates and equivalent isotropic displacement parameters for C_6F_5Cl (III) at 150 K. U_{eq} is defined as ⅓ of the trace of the orthogonalised U_{ij} tensor.

Atom	*x*	*y*	*z*	*U*(eq) / Å²
Cl(1)	0.23659(6)	0	0.06092(7)	0.0381(3)
F(1)	−0.22267(15)	0	0.44435(17)	0.0440(4)
F(2)	−0.11364(12)	0.3063(14)	0.35763(14)	0.0432(3)
F(3)	0.5980(11)	0.3074(13)	0.17847(13)	0.0399(3)
C(1)	−0.1168(2)	0	0.3570(3)	0.0297(5)
C(2)	−0.06211(17)	0.1561(2)	0.31181(19)	0.0287(4)
C(3)	0.04716(17)	0.1552(2)	0.22167(18)	0.0270(4)
C(4)	0.1026(2)	0	0.1747(2)	0.0259(5)

Table S3c. Anisotropic displacement parameters for C_6F_5Cl (III) at 150 K. The anisotropic displacement factor exponent has the form: $-2\pi^2[h^2a^{*2}U_{11}+2hka^*b^*U_{12}+\ldots]$.

Atom	U_{11} / Å²	U_{22} / Å²	U_{33} / Å²	U_{23} / Å²	U_{13} / Å²	U_{12} / Å²
Cl(1)	0.0260(3)	0.0563(4)	0.0341(4)	0	0.0127(2)	0
F(1)	0.0307(7)	0.0671(11)	0.0374(9)	0	0.0183(6)	0
F(2)	0.0415(6)	0.0366(6)	0.0519(7)	−0.0114(5)	0.0071(5)	0.0131(4)
F(3)	0.0391(6)	0.0279(5)	0.0531(7)	0.0090(4)	0.0073(5)	−0.0052(4)
C(1)	0.0190(9)	0.0427(13)	0.0282(12)	0	0.0068(8)	0
C(2)	0.0243(7)	0.0284(8)	0.0329(9)	−0.0041(6)	0.0017(6)	0.0052(6)
C(3)	0.0224(7)	0.0268(8)	0.0315(9)	0.0029(6)	0.0015(6)	−0.0021(5)
C(4)	0.0179(9)	0.0316(11)	0.0282(12)	0	0.0031(8)	0

Table S3d. Selected bond lengths for C_6F_5Cl (III) at 150 K.

Atom — Atom	Length / Å	Atom — Atom	Length / Å
Cl(1) — C(4)	1.711(2)	C(1) — C(2)	1.380(2)
F(1) — C(1)	1.334(3)	C(2) — C(3)	1.377(3)
F(2) — C(2)	1.3318(18)	C(3) — C(4)	1.383(2)
F(3) — C(3)	1.3344(18)	C(4) — C(3)[1]	1.383(2)
C(1) — C(2)[1]	1.380(2)		

[1] *x*, 1−*y*, *z*

Table S4. Table of lattice parameters obtained from LeBail refinements to the two PXRD data sets shown in Figs. S1 and S2. No unit cell is available for phase I. Percentage molecular volume changes are shown in Fig. 5 for the second set of measurements; points from the first set of data would almost superimpose. The tiny differences in the values obtained from the two data sets is attributable to small systematic errors. The latter are due to the result of performing LeBail fits on data suffering from the effects of sample granularity.

T / K	Phase	S.G.	*Z*	*a* / Å	*b* / Å	*c* / Å	α / °	β / °	γ / °	*V* / *Z* / Å³
90	III	*C*2/*m*	4	8.9806(7)	7.6358(5)	9.4241(7)	90	96.962(5)	90	160.37(2)
100	III	*C*2/*m*	4	8.9886(8)	7.6405(5)	9.4385(7)	90	96.887(5)	90	160.89(2)
110	III	*C*2/*m*	4	8.9901(8)	7.6416(6)	9.4429(7)	90	96.839(5)	90	161.03(2)
120	III	*C*2/*m*	4	8.9953(7)	7.6440(5)	9.4529(7)	90	96.775(4)	90	161.36(2)
130	III	*C*2/*m*	4	8.9999(7)	7.6478(5)	9.4658(7)	90	96.722(5)	90	161.76(2)
140	III	*C*2/*m*	4	9.0055(7)	7.6501(5)	9.4775(7)	90	96.656(4)	90	162.13(2)
150	III	*C*2/*m*	4	9.0130(7)	7.6550(5)	9.4922(7)	90	96.587(5)	90	162.65(2)
160	III	*C*2/*m*	4	9.0173(7)	7.6602(5)	9.5068(7)	90	96.524(5)	90	163.11(2)
170	III	*C*2/*m*	4	9.0266(8)	7.6660(5)	9.5268(7)	90	96.432(6)	90	163.77(2)
180	III	*C*2/*m*	4	9.0393(7)	7.6767(5)	9.5502(8)	90	96.327(7)	90	164.67(2)
190	II	*C*2/*m*	2	9.0844(9)	7.7308(6)	4.7943(4)	90	96.225(5)	90	167.36(2)
200	II	*C*2/*m*	2	9.1099(9)	7.7688(7)	4.8005(4)	90	95.979(6)	90	168.95(3)
210	II	*C*2/*m*	2	9.1271(9)	7.8086(6)	4.8010(4)	90	95.683(5)	90	170.24(3)
220	II	*C*2/*m*	2	9.1440(10)	7.8514(7)	4.8052(4)	90	95.397(6)	90	171.72(3)
230	II	*C*2/*m*	2	9.1496(10)	7.8836(8)	4.8146(4)	90	95.164(6)	90	172.94(3)
240	II	*C*2/*m*	2	9.1468(10)	7.9100(9)	4.8322(4)	90	94.956(6)	90	174.16(3)
250	I	?	?	?	?	?	?	?	?	?
100	III	*C*2/*m*	4	8.9857(6)	7.6444(4)	9.4297(5)	90	96.956(4)	90	160.74(2)
110	III	*C*2/*m*	4	8.9953(4)	7.6477(4)	9.4438(5)	90	96.898(3)	90	161.24(1)
120	III	*C*2/*m*	4	8.9995(5)	7.6523(4)	9.4555(4)	90	96.819(3)	90	161.64(1)
130	III	*C*2/*m*	4	9.0045(5)	7.6556(4)	9.4675(4)	90	96.754(4)	90	162.03(1)
140	III	*C*2/*m*	4	9.0139(5)	7.6585(4)	9.4791(4)	90	96.696(4)	90	162.48(1)
150	III	*C*2/*m*	4	9.0176(5)	7.6619(4)	9.4938(4)	90	96.619(3)	90	162.89(1)
160	III	*C*2/*m*	4	9.0263(5)	7.6673(4)	9.5088(4)	90	96.548(3)	90	163.45(1)
170	III	*C*2/*m*	4	9.0315(5)	7.6727(4)	9.5262(5)	90	96.482(4)	90	163.98(2)
180	III	*C*2/*m*	4	9.0424(5)	7.6833(4)	9.5464(5)	90	96.398(4)	90	164.78(2)
190	II	*C*2/*m*	2	9.0889(7)	7.7358(5)	4.7931(3)	90	96.264(5)	90	167.49(2)
200	II	*C*2/*m*	2	9.1107(7)	7.7739(5)	4.7986(2)	90	96.025(5)	90	168.99(2)
210	II	*C*2/*m*	2	9.1294(8)	7.8167(5)	4.8002(3)	90	95.718(6)	90	170.42(2)
220	II	*C*2/*m*	2	9.1447(7)	7.8560(5)	4.8034(2)	90	95.433(5)	90	171.76(2)
230	II	*C*2/*m*	2	9.1481(7)	7.8890(5)	4.8128(3)	90	95.170(5)	90	172.96(2)
240	II	*C*2/*m*	2	9.1406(10)	7.9189(6)	4.8298(3)	90	94.941(7)	90	174.15(2)
250	II	*C*2/*m*	2	9.1254(8)	7.9413(6)	4.8558(3)	90	94.752(6)	90	175.34(2)

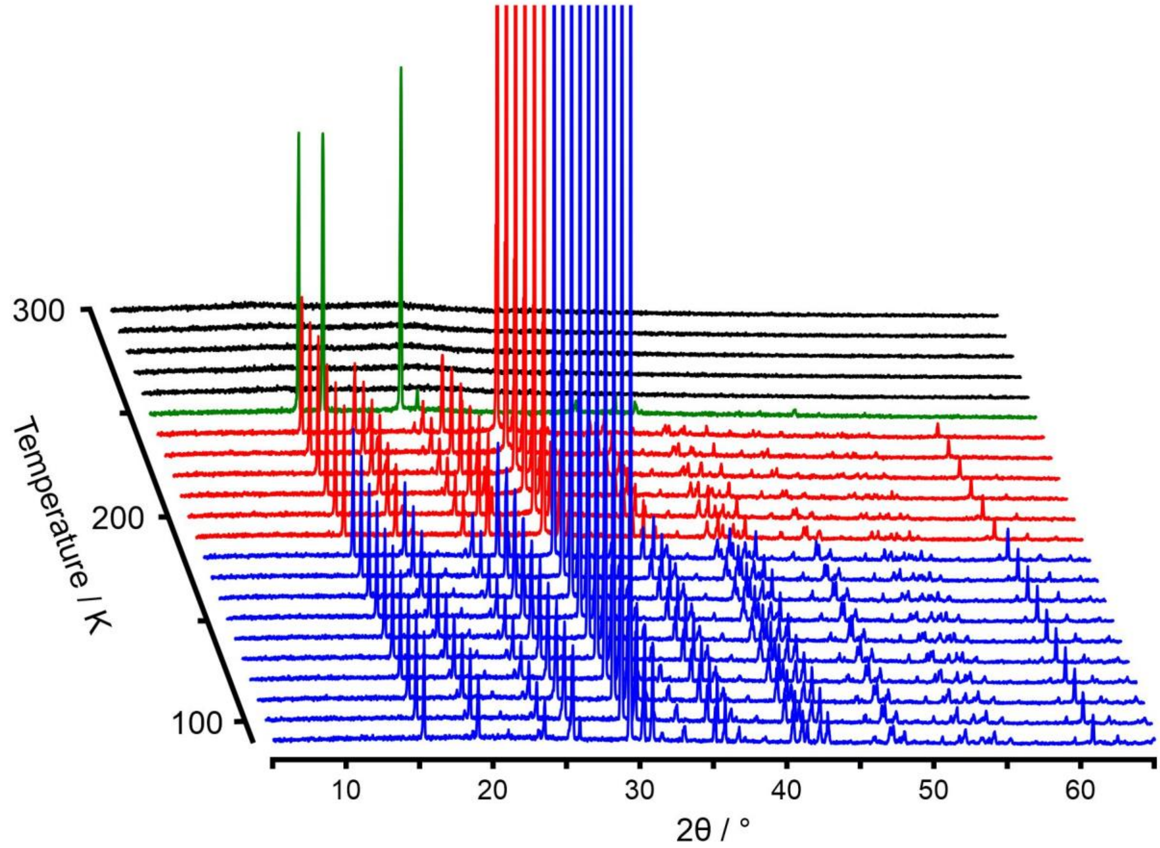

Fig. S1. VT-PXRD data on C_6F_5Cl measured in a 1.0 mm X-ray capillary on a Stoe Stadi-P diffractometer with Cu Kα_1 radiation as a function of temperature on heating. Phase III is shown in blue, phase II in red, and the elusive phase I in green (with the melt shown in black). The lower temperature patterns in blue have additional weaker peaks relative to the patterns shown in red due to the doubled-size unit cell exhibited by phase III.

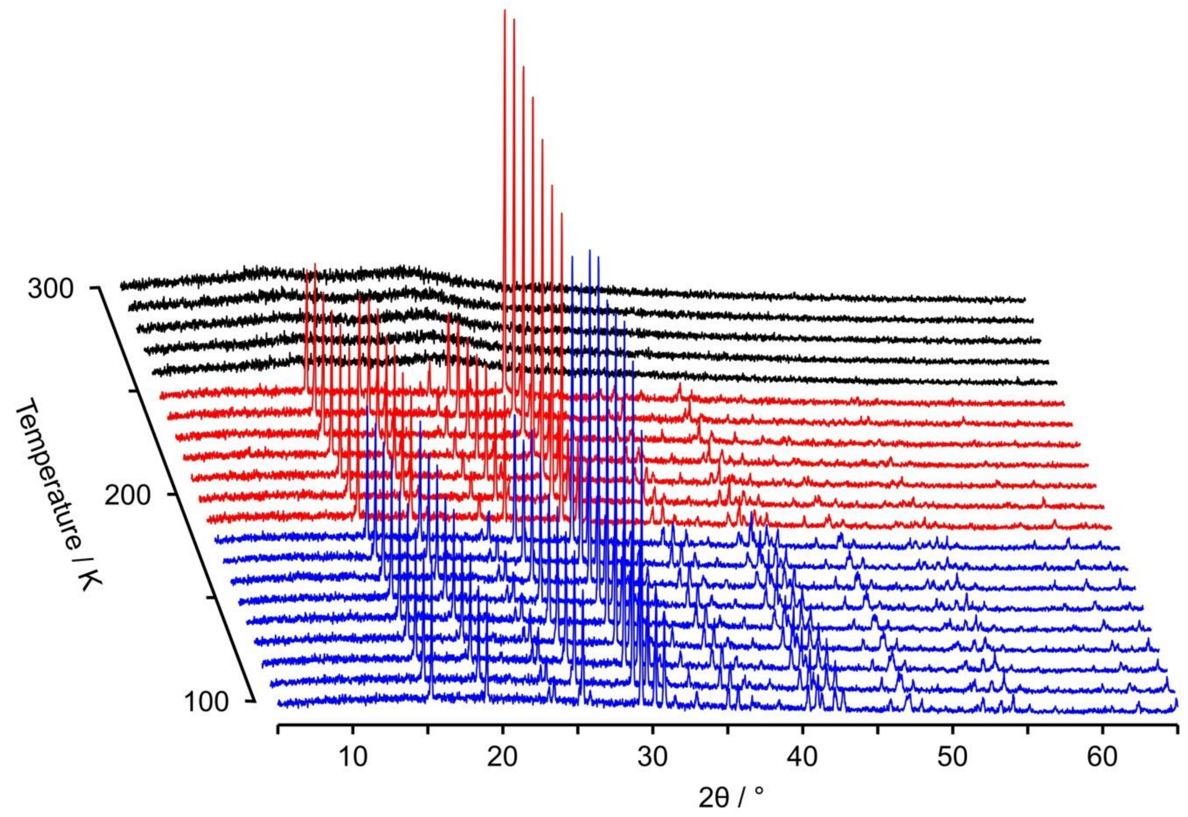

Fig. S2. VT-PXRD data on C_6F_5Cl measured in a 1.0 mm X-ray capillary on a Stoe Stadi-P diffractometer with Cu Kα_1 radiation as a function of temperature on heating. The colours are the same as for Fig. S1. Notice that phase I is absent from this set of measurements.

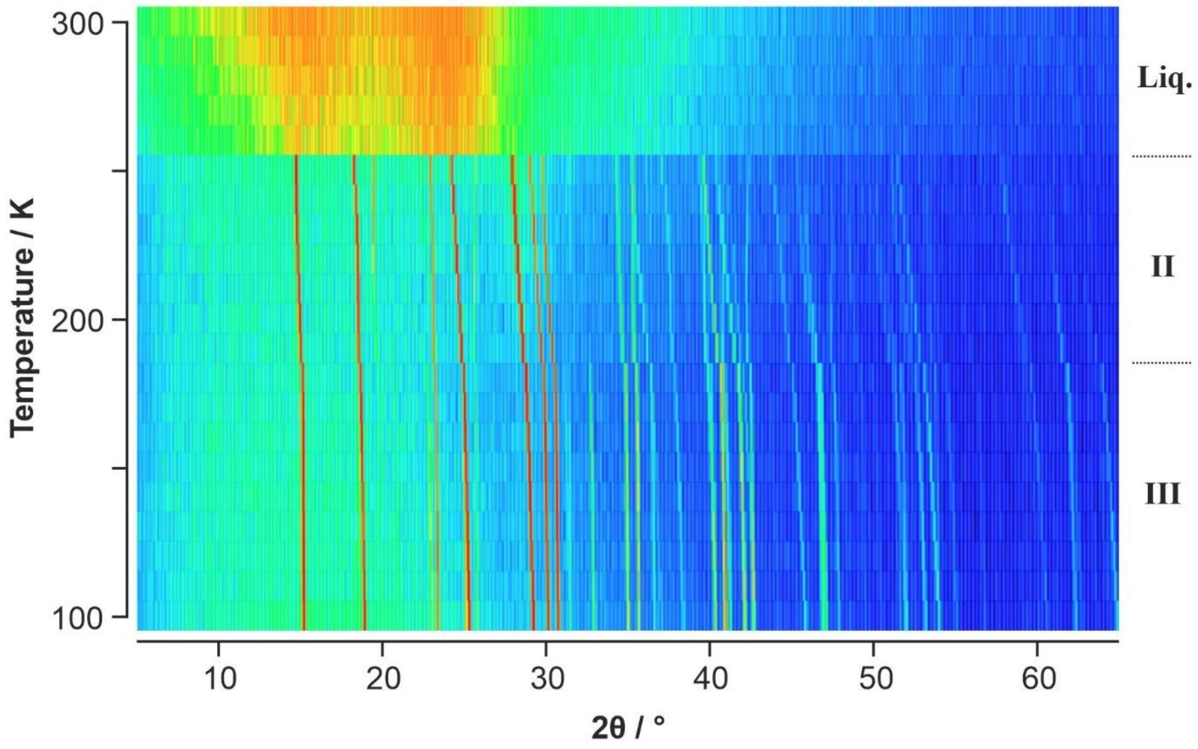

Fig. S3. The same VT-PXRD data as for Fig. S2, but now shown as a surface colour plot where the colour scale shows low intensities in the PXRD patterns in blue, intermediate intensities are shown in green/yellow, and high intensities in orange/red.

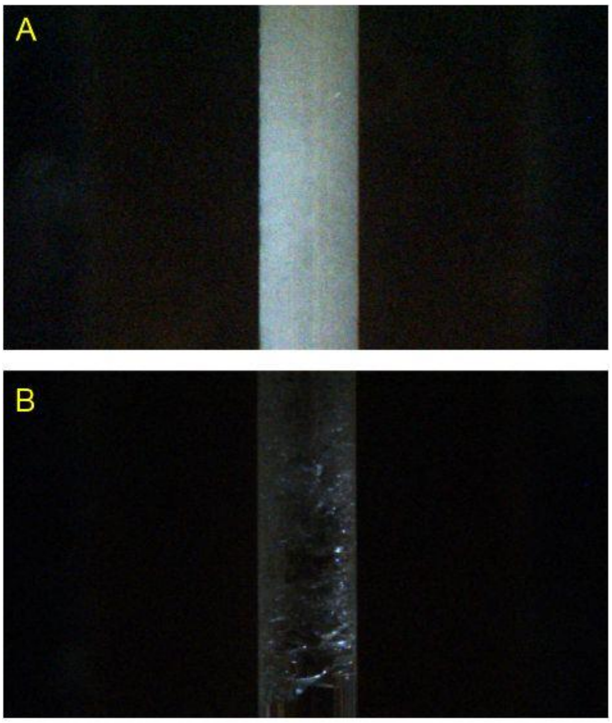

Fig. S4. Capillary (nominal Ø 1 mm) used in the VT-PXRD study containing C_6F_5Cl. **A** (*top*) shows the appearance of the sample when flash-frozen; and **B** (*bottom*) shows the same sample slow cooled from the liquid and then reheated to just below the melt (250 K).

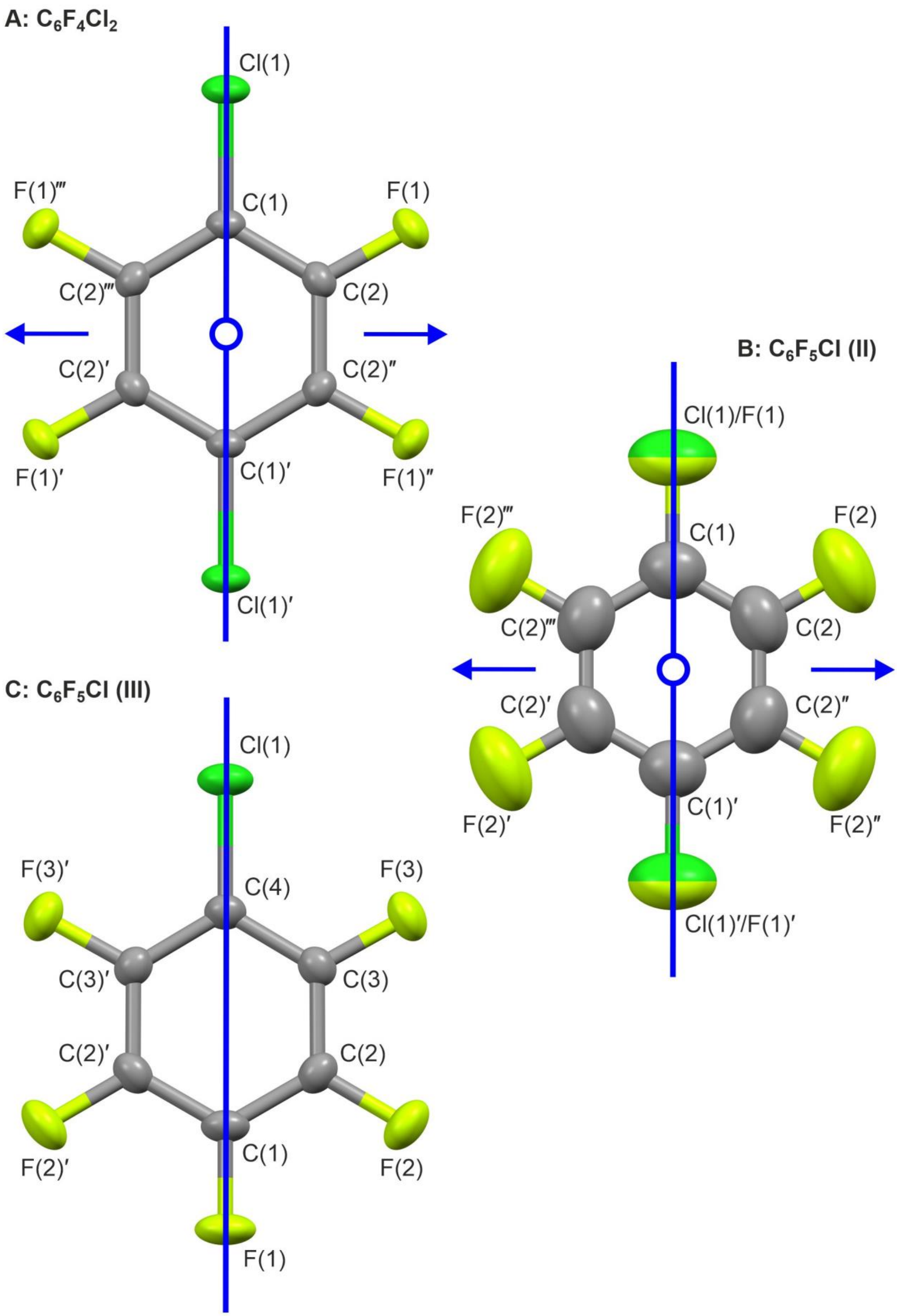

Fig. S5. Labels of the atoms used to describe the crystal structures of *p*-$C_6F_4Cl_2$ **(a)** and the two solid-state phases of C_6F_5Cl **(b)** and **(c)** shown in Fig. 3. Point-group symmetry elements are shown in blue. The prime symbols on labels indicates symmetry-related atoms.

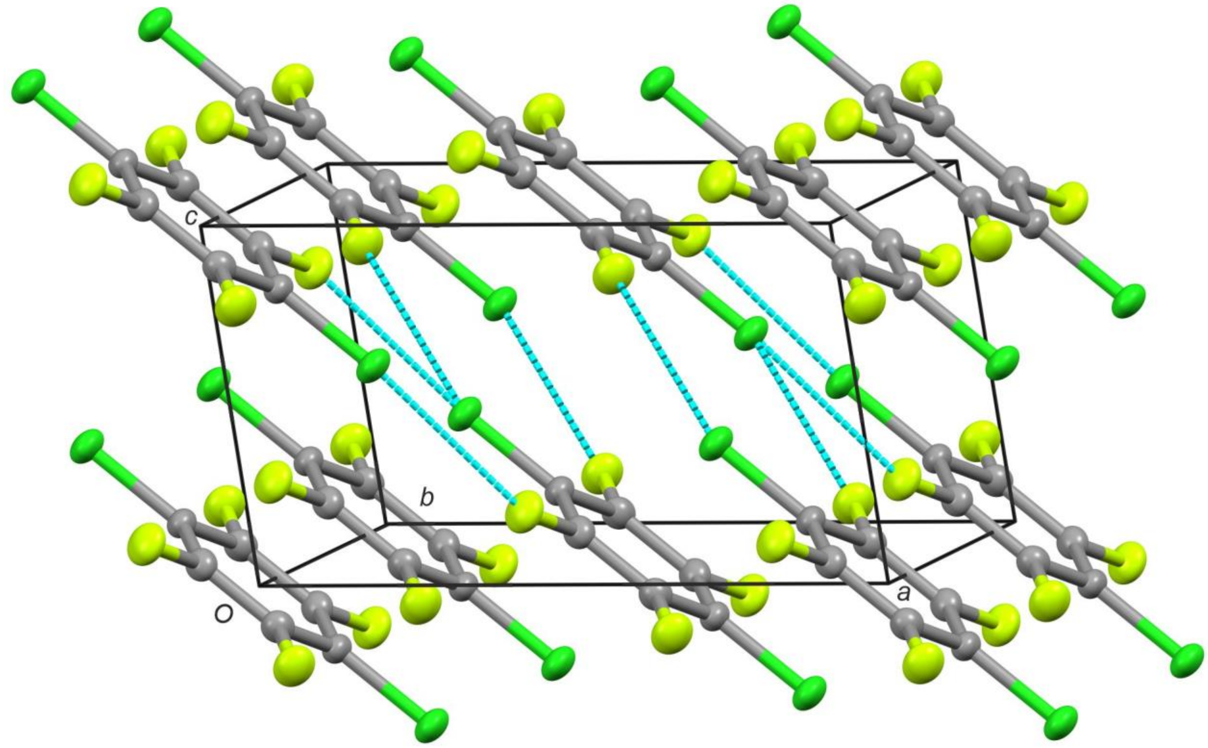

Fig. S6. Unit cell of p-$C_6F_4Cl_2$ (C = grey, F = light green, and Cl = green) showing the structure at 150 K. Thermal ellipsoids are drawn at 50% probability using Mercury.[5] The closest F···Cl contacts, all equal to 3.19 Å, are illustrated with dashed cyan sticks. Closest Cl···π(C_6-ring) contacts are shown in Fig. S7. The molecules are arranged in parallel sheets (see Fig. S8) and not in a classic herringbone structure as exhibited, for example, by benzene.

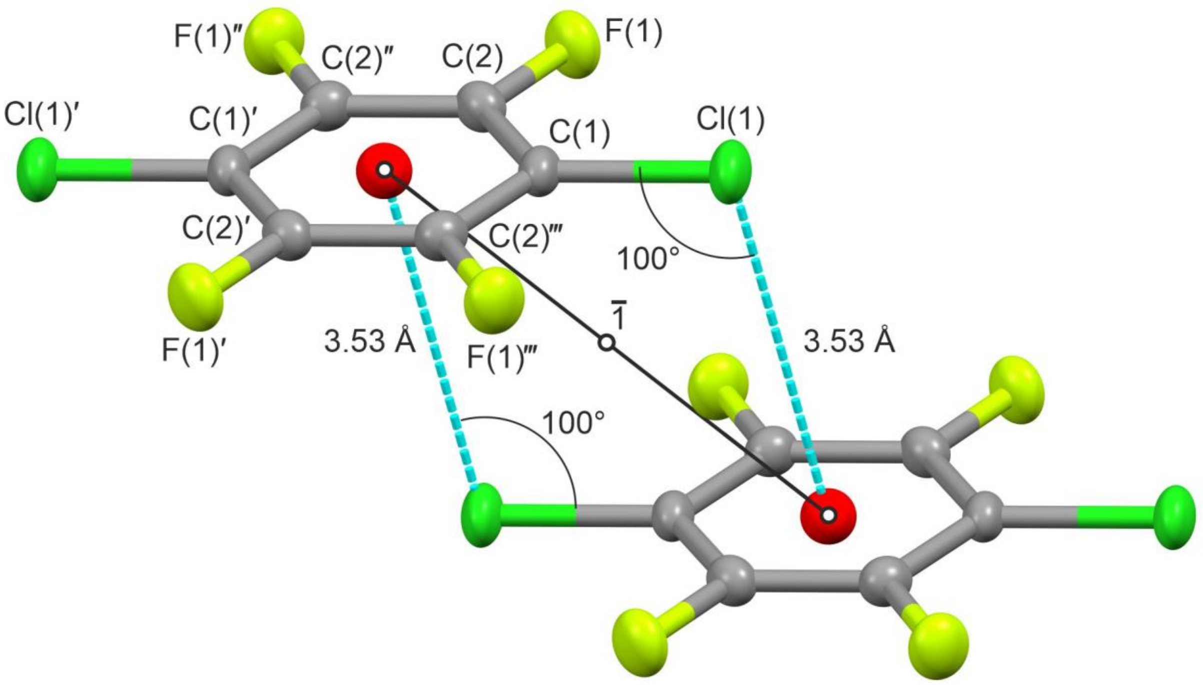

Fig. S7. Closest Cl···π(C_6-ring) contacts and inversion symmetry between adjacent interacting molecules in the structure of p-$C_6F_4Cl_2$ at 150 K. Centre of mass of each molecule is shown with red spheres.

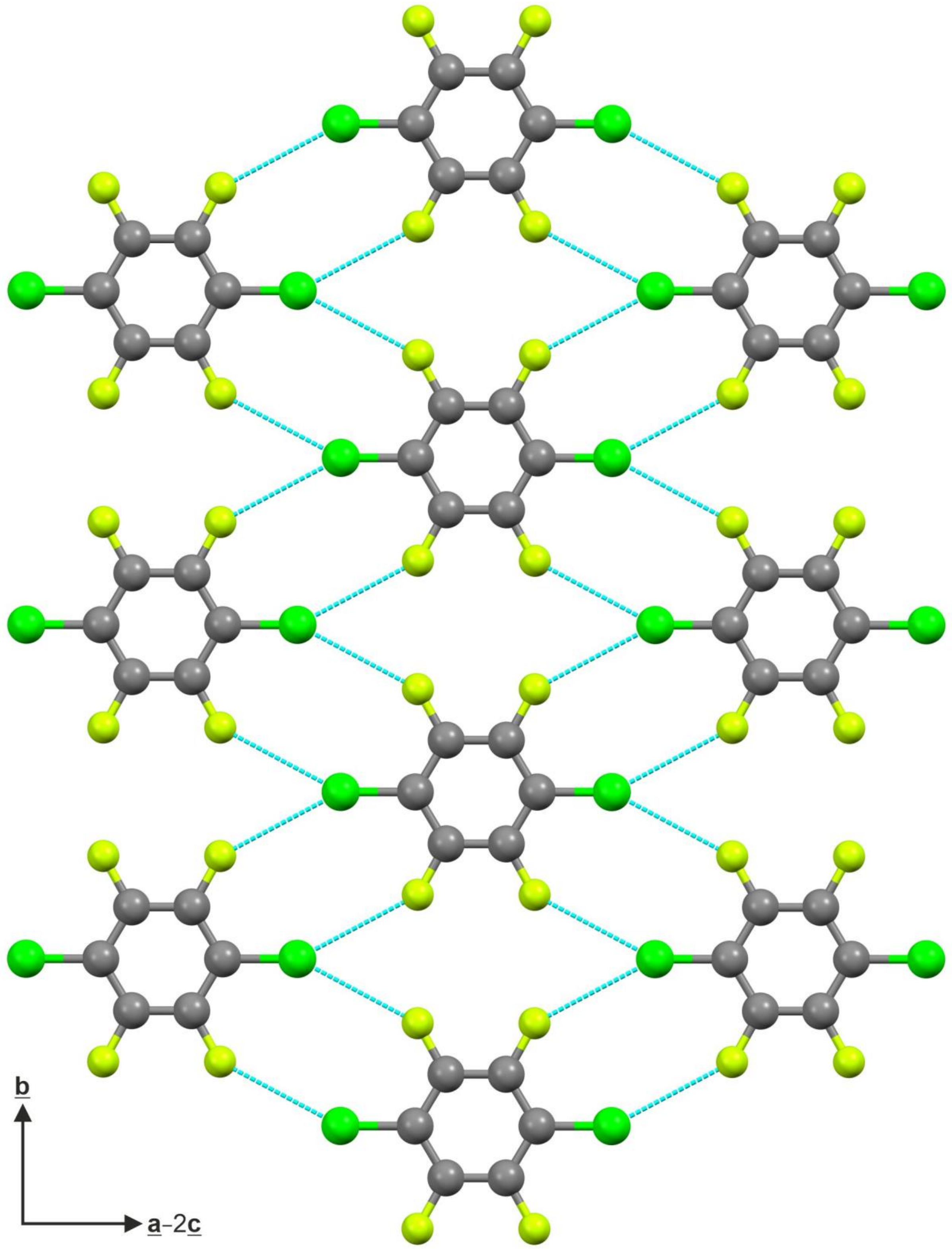

Fig. S8. Flat sheet of *p*-$C_6F_4Cl_2$ molecules (shown as "ball & stick") with the network of closest F···Cl contacts, all equal to 3.19 Å, illustrated with dashed cyan sticks. All molecules lie approximately in the same plane. The interaction of *p*-$C_6F_4Cl_2$ molecules from one plane to the next is illustrated in Fig. S7. Similar sheets of molecules are observed in the isomorphous phase II of C_6F_5Cl, as well as in C_6F_5Cl in phase III (see Fig. S9).

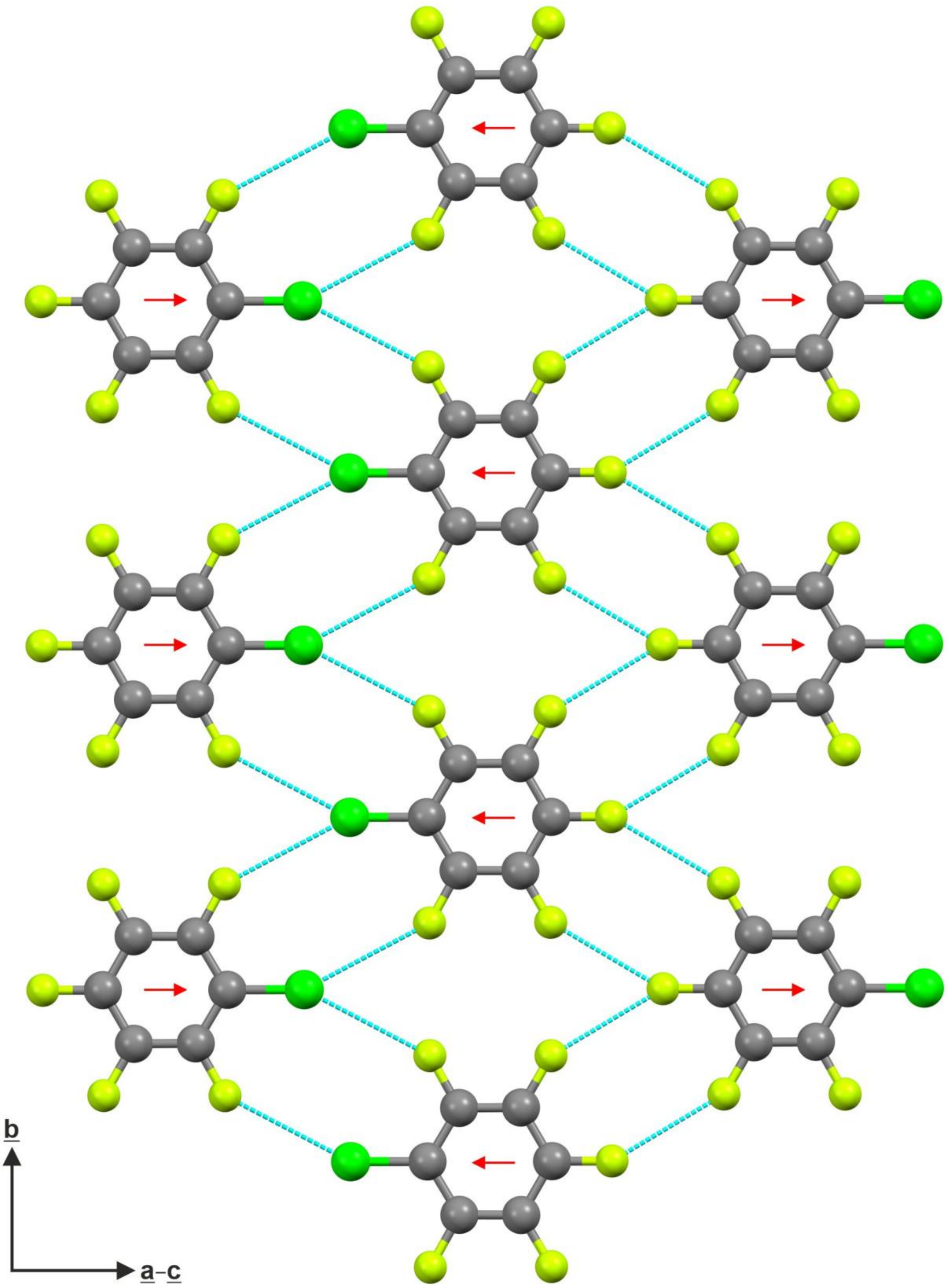

Fig. S9. Flat sheet of C_6F_5Cl molecules in its antiferroelectric phase III (shown as "ball & stick") with the network of closest F···F and F···Cl contacts, equal to 2.92 Å and 3.19 Å, respectively, illustrated with dashed cyan sticks. All molecules lie almost in the same plane, with the molecular electric dipole moments (shown in red) aligned perpendicular both to **b** and approximately co-linear with **a**−**c**.

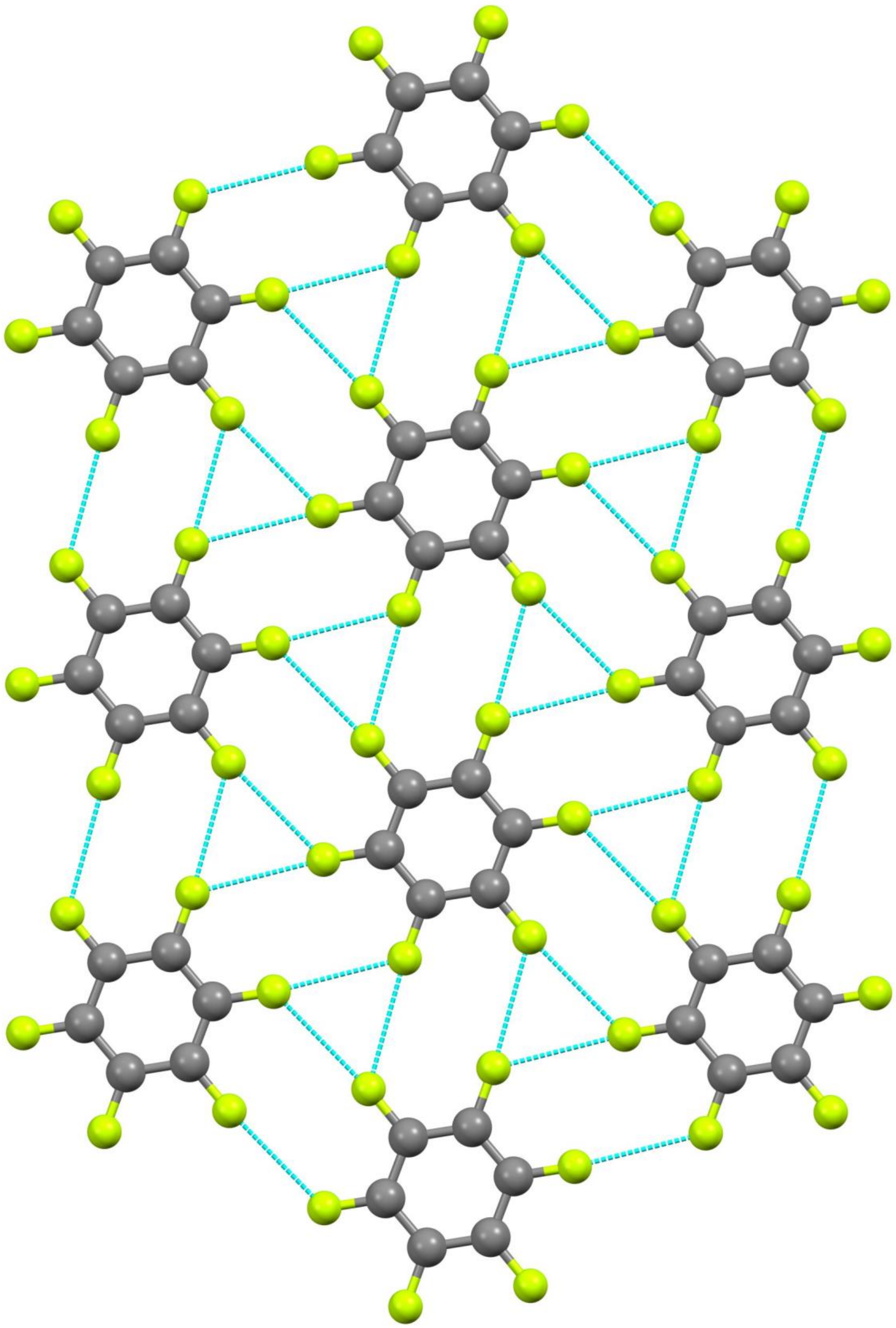

Fig. S10. Flat sheet of C_6F_6 molecules in its high-pressure phase II (shown as "ball & stick") redrawn from CCDC entry HFBENZ15. All molecules lie approximately in the same plane in marked contrast to the arrangement of the molecules in C_6F_6 phase I. In contrast to *p*-$C_6F_4Cl_2$ (Fig. S8) and C_6F_5Cl (Fig. S9), a rotation of the C_6F_6 molecules about the normal to the C_6-ring leads to the absence of any mirror symmetry in this structure.

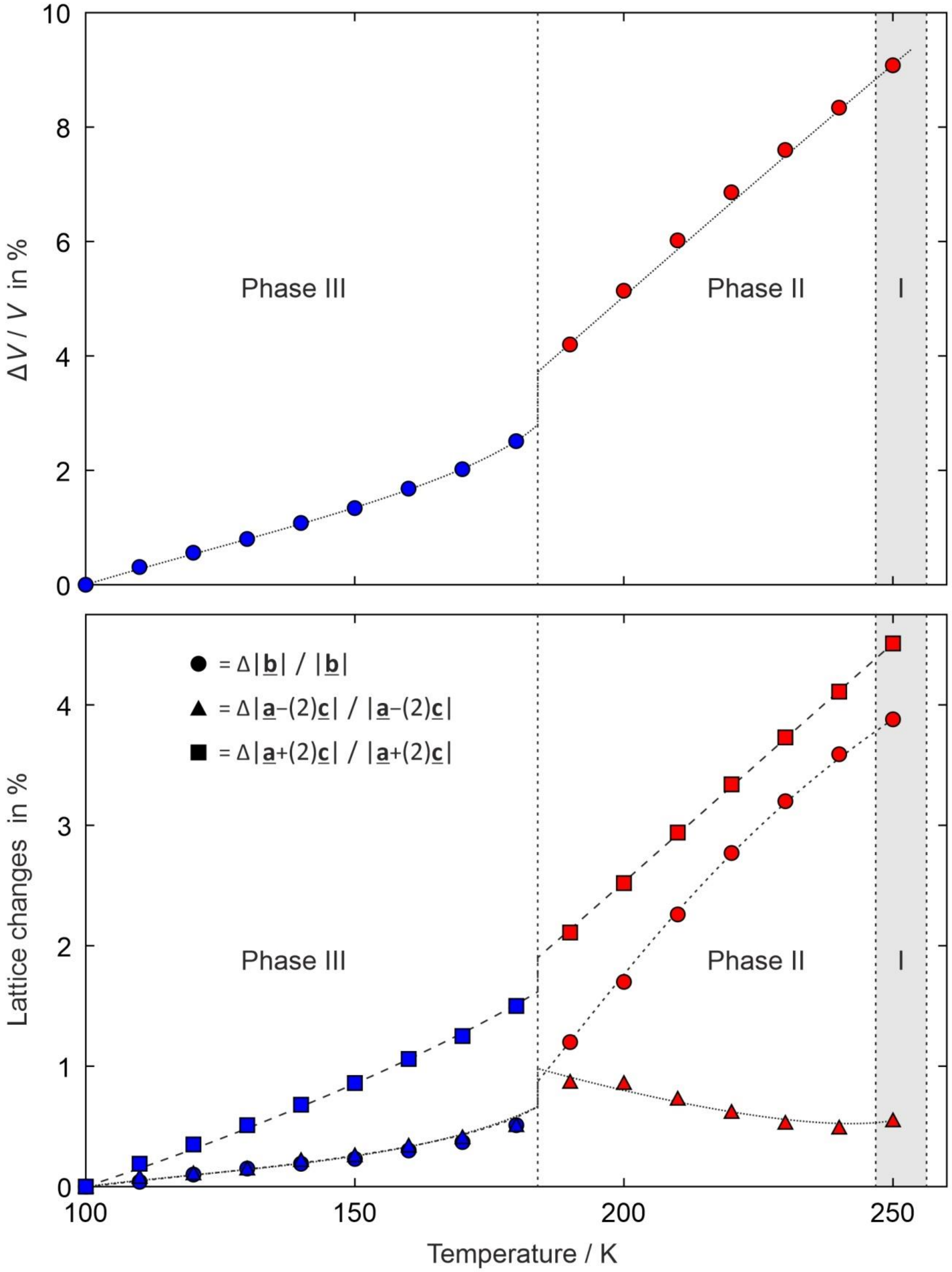

Fig. S11. (*Top*): Percentage change in molecular volume of C_6F_5Cl as a function of temperature (relative to 100 K) showing the step change in volume between phases II (points in red) and III (points in blue). The dotted lines linking the points are a guide to the eye. Numerical values for the data points (obtained from the results of LeBail fits to the raw VT-PXRD data) are available in Table S4. The grey area indicates the region where earlier DSC had reported an additional phase as seen by us under certain conditions in PXRD. (*Bottom*): Percentage lattice changes (relative to 100 K) in the thermal expansion of C_6F_5Cl with respect to the directions **b**, **a**−**c**, and **a**+**c** (for phase III), and **b**, **a**−2**c**, and **a**+2**c** (as *c* in phase II is halved relative to phase III). These values are derived readily from the values of *a*, *c*, and β given in Table S4 using the cosine rule.

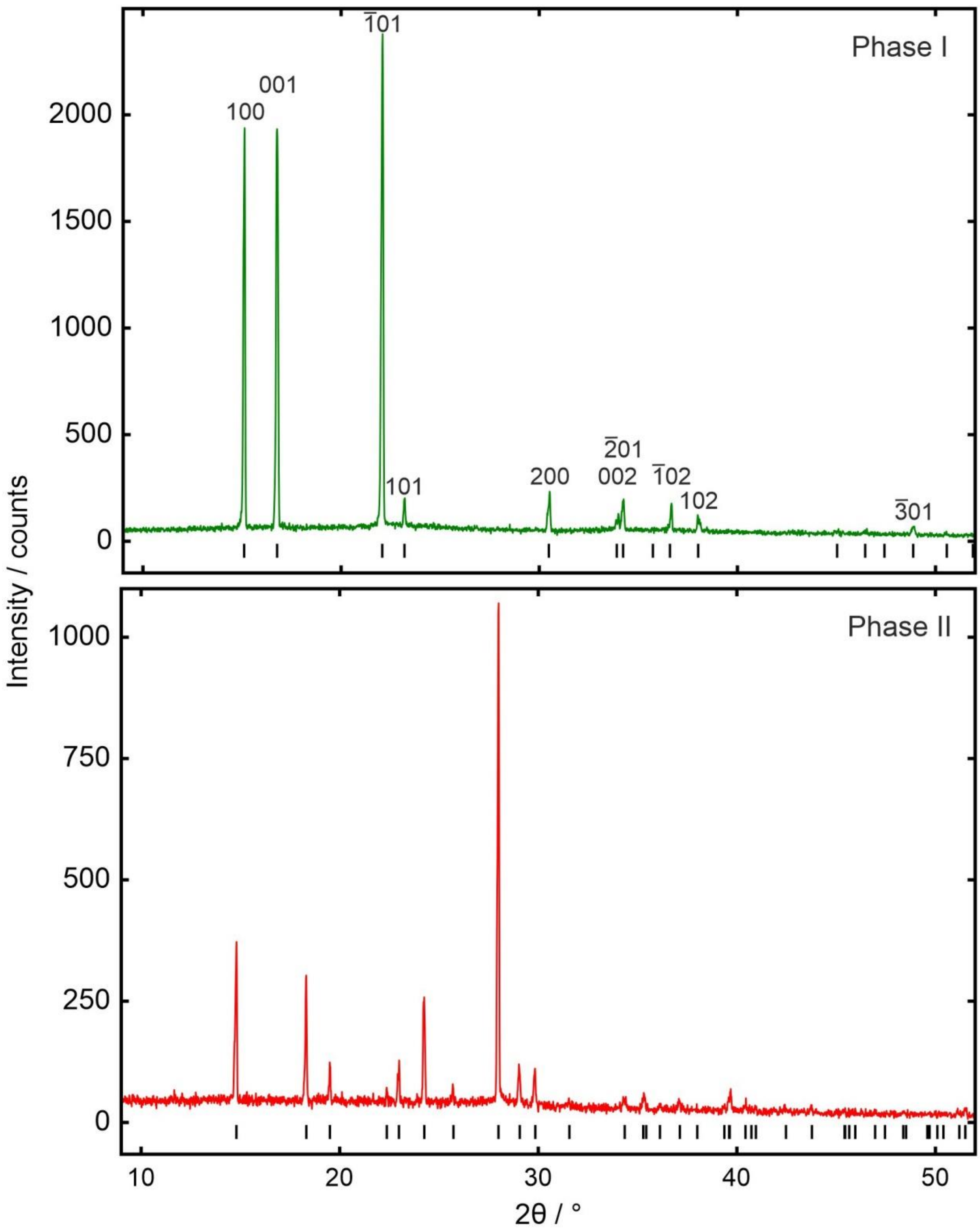

Fig. S12. PXRD data on phases I (*top*) and II (*bottom*) of C_6F_5Cl at 250 K. Indexing of phase II matches the unit cell determined from the SXD studies. Visible peaks in phase I may be indexed as *h*0*l* reflections as shown where the tick marks are calculated for a 2-D lattice with a = 5.867 Å, c = 5.289 Å, and β = 92.81°.

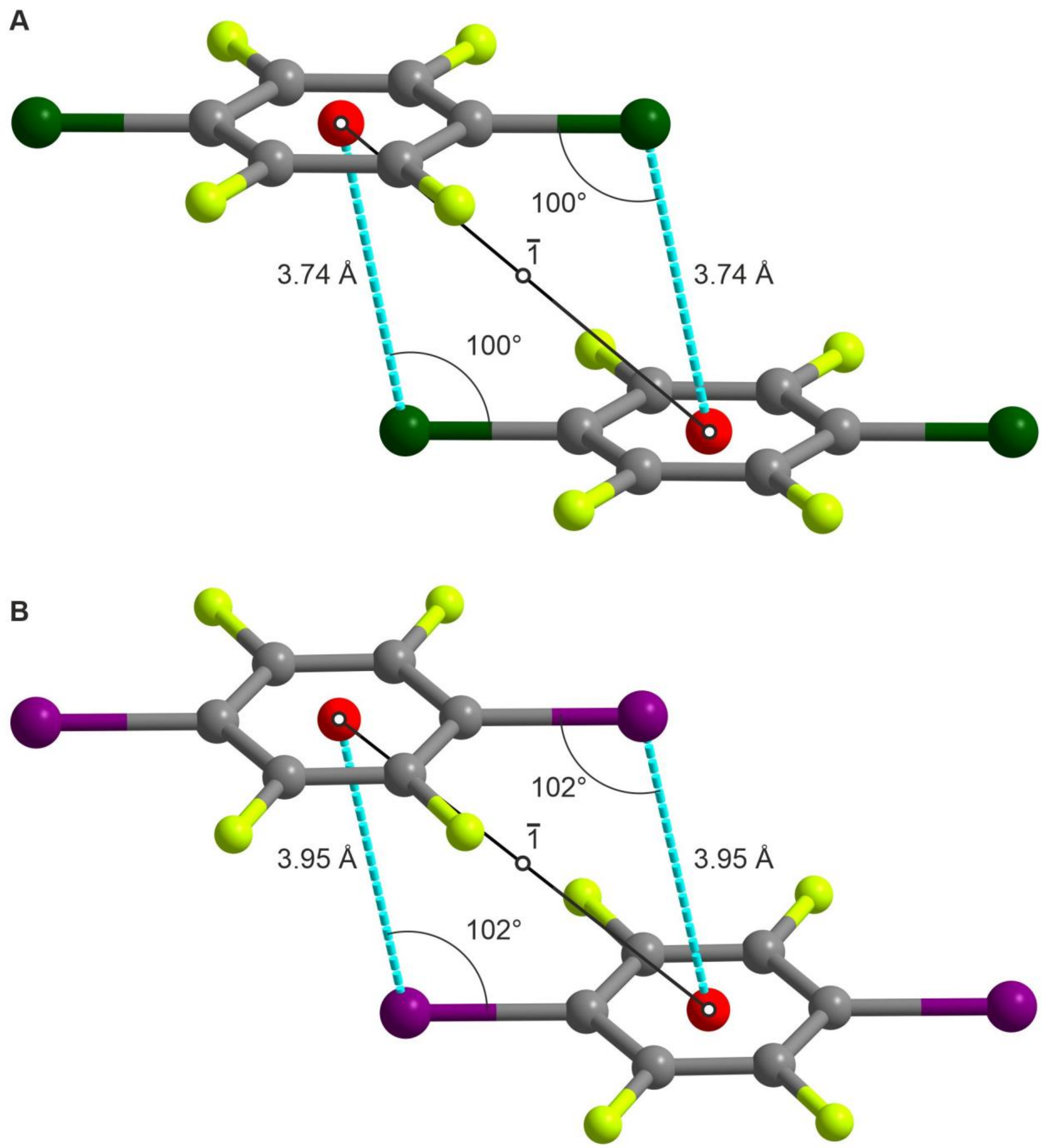

Fig. S13. Closest X···π(C_6-ring) contacts between adjacent interacting molecules in the structures of **A** p-$C_6F_4Br_2$ at 100 K and **B** p-$C_6F_4I_2$ at 180 K drawn from the CIF data from references 31 and 32 respectively. Atoms are coloured as follows: C (grey), F (light green), Br (dark green), and I (purple). The motif is identical to that seen in the form of p-C_6F_4Cl reported here, as shown in Figs. 4 and S7. Crystallographic inversion symmetry is shown with black open circles and the centre of mass of each molecule is shown with red spheres. However, in marked contrast to p-$C_6F_4Cl_2$, the molecules do not lie in sheets as in Fig. S8.

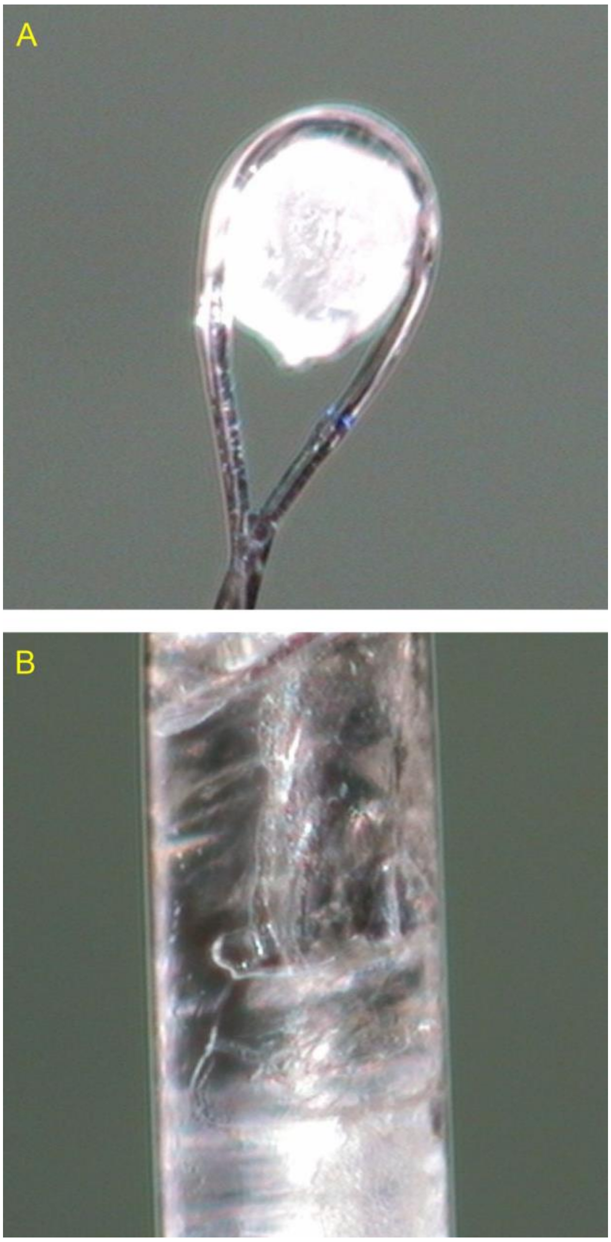

Fig. S14. Photograph of the samples measured by SXD in this study: **A** (*top*) is a crystal of *p*-$C_6F_4Cl_2$ on a 20 µm nylon loop (nominal Ø 0.3 mm) and **B** (*bottom*) is solid C_6F_5Cl in a 0.3 mm (nominal Ø) capillary.
